# Colonization by *B. infantis* EVC001 modulates enteric inflammation in exclusively breastfed infants

**DOI:** 10.1038/s41390-019-0533-2

**Published:** 2019-08-23

**Authors:** Bethany M. Henrick, Stephanie Chew, Giorgio Casaburi, Heather K. Brown, Steven A. Frese, You Zhou, Mark A. Underwood, Jennifer T. Smilowitz

**Affiliations:** 1 Evolve Biosystems, Inc, Davis, CA USA; 2 Department of Food Science and Technology, University of Nebraska, Lincoln, NE USA; 3 Morrison Microscopy Core Research Facility, University of Nebraska, Lincoln, NE USA; 4 Foods for Health Institute, University of California Davis, Davis, CA USA; 5 Department of Pediatrics, University of California Davis Children’s Hospital, Sacramento, CA USA; 6 Department of Food Science and Technology, University of California Davis, Davis, CA USA

## Abstract

**Background:**

Infant gut dysbiosis, often associated with low abundance of bifidobacteria, is linked to impaired immune development and inflammation—a risk factor for increased incidence of several childhood diseases. We investigated the impact of *B. infantis* EVC001 colonization on enteric inflammation in a subset of exclusively breastfed term infants from a larger clinical study.

**Methods:**

Stool samples (*n* = 120) were collected from infants randomly selected to receive either 1.8 × 10^10^ CFU *B. infantis* EVC001 daily for 21 days (EVC001) or breast milk alone (controls), starting at day 7 postnatal. The fecal microbiome was analyzed using 16S ribosomal RNA, proinflammatory cytokines using multiplexed immunoassay, and fecal calprotectin using ELISA at three time points: days 6 (Baseline), 40, and 60 postnatal.

**Results:**

Fecal calprotectin concentration negatively correlated with *Bifidobacterium* abundance (*P <* 0.0001; *ρ* = −0.72), and proinflammatory cytokines correlated with *Clostridiaceae* and *Enterobacteriaceae*, yet negatively correlated with *Bifidobacteriaceae* abundance. Proinflammatory cytokines were significantly lower in EVC001-fed infants on days 40 and 60 postnatally compared to baseline and compared to control infants.

**Conclusion:**

Our findings indicate that gut dysbiosis (absence of *B. infantis*) is associated with increased intestinal inflammation. Early addition of EVC001 to diet represents a novel strategy to prevent enteric inflammation during a critical developmental phase.

## Introduction

The intestinal microbiome plays a critically important role in the maturation of the immune system, especially during the first 100 days of life.^1–3^ Moreover, recent reports suggest that a dysbiotic microbiome leading to aberrant, proinflammatory responses in early life can have numerous long-term health consequences.^1–3^ Specifically, microbial gut dysbiosis during infancy has been linked to increased risk of developing acute and long-term inflammatory diseases, such as colic,^4^ asthma and allergies,^3,5^ type 1 diabetes,^6,7^ obesity,^8^ and celiac disease.^9^ This is supported by mechanistic and observational studies, which provide strong evidence that alterations in the gut microbiome composition and metabolism contribute to immune disruptions. In particular, disruption of immune homeostasis early in life can lead to greater risk of the onset and progression of various autoimmune and allergic diseases.^3,10,11^ Recently, a longitudinal study of newborn infants using systems-level immune analyses found that gut dysbiosis in infants is associated with increased circulating endothelial cells, activated effector T cells, and inflammatory cytokine production.^1^

Recent reports have shown that early gut dysbiosis in infants induces enteric inflammation, as exhibited by the increased expression of fecal calprotectin,^4,12^ an antimicrobial protein released from infiltrating neutrophils and mucosal macrophages during inflammatory conditions.^13^ Indeed, increased fecal calprotectin has been previously identified in preterm infants suffering from intestinal distress, enteropathies, and necrotizing enterocolitis (NEC)^12,14–16^ and term infants with colic.^4^

Evaluation of systemic chronic inflammation in infants has identified that several cytokines (e.g., interleukin (IL)-6, IL-17, IL-22, tumor necrosis factor-α (TNFα), and interferon-γ (IFNγ)) are instrumental in the progression of autoimmune and allergic diseases;^1,3,17–19^ however, few reports describe chronic intestinal inflammation in infant populations, which is important given the association with the underlying pathogenesis of autoimmune disease in adults.^20^ Moreover, the production of such cytokines has been well established to impair gut barrier function, leading to increased intestinal permeability, which has both short- and long-term consequences, including eczema, Crohn’s disease, ulcerative colitis, and type 1 diabetes,^21–23^ as well as drives immunopathogenesis.^24^

Importantly, an increasing number of reports in animal models, infants, and children have connected host health outcomes to specific gut microbes and demonstrated how differential microbiome compositions may influence immune system development.^3,25^ Indeed, disruption of the early gut microbiome composition,^3^ exposure to antibiotics,^10^ and an abundance of *Proteobacteria* in early life are linked to immune dysregulation later in life.^2,26^ Conversely, the abundance of *Bifidobacterium*, specifically *Bifidobacterium longum* subsp. *infantis* (*B*. *infantis*), has been shown to negatively correlate with *Proteobacteria*^7,26,27^ and a number of negative health outcomes.^12,26,27^ Recent publications have highlighted that gut dysbiosis and low *B. infantis* abundance at 2 months of age strongly correlates with significantly decreased vaccine responses at 6 months and 2 years compared to infants not colonized by *B. infantis*.^28^

While several seminal publications have revealed the evolution of *B. infantis* as the predominant strain colonizing the breastfed infant microbiome due to its unique ability to consume human milk oligosaccharides (HMOs),^27,29^ recent cohort studies indicate that *B. infantis* is far less abundant in infants born in industrialized nations compared to reports from developing nations.^27,28,30^ This organism is the predominant subspecies found in breastfed infants from resource-limited areas, where *B. infantis* is abundant among healthy, breastfed infants. Historical evidence suggests this organism has been lost over the past century, and its absence is associated with an increased abundance of *Proteobacteria*, a hallmark of dysbiosis.^26,27,30^ Moreover, previous publications have shown infants with high levels of *Proteobacteria* display decreased production of short-chain fatty acids, reduced utilization of HMOs, increased degradation of colonic mucin, and increased fecal pH, as well as elevated concentrations of fecal endotoxin compared to infants who exhibited a *B. infantis*-dominated gut microbiome.^27,30,31^

Given recent work identifying degradation in the colonic mucin layer and significantly increased fecal endotoxin levels in infants who lack *B. infantis*,^27,31^ as well as new evidence highlighting the importance of the first 100 days of life in shaping immune development in infants,^1,3^ we hypothesized that feeding *B. infantis* EVC001 to exclusively breastfed infants would also result in decreased enteric inflammation. To test this hypothesis, we evaluated fecal markers of inflammation, including calprotectin and cytokine production, in samples collected over the first 60 days postnatal from exclusively breastfed infants fed *B. infantis* EVC001 compared to infants consuming breast milk alone.

## Materials and methods

### Study design

A total of 120 fecal samples (ClinicalTrials.gov: NCT02457338; https://clinicaltrials.gov/ct2/show/NCT02457338) from 40 different subjects were analyzed at three time points—day 6 (Baseline), day 40, and day 60 postnatally. Individual subjects were chosen at random and made up a subset of the original study participants. All aspects of the study were approved by the University of California Davis Institutional Review Board (IRB Number: ID 631099) and all participants provided written informed consent. Details of the study design and procedures used to collect these samples can be found in ref. ^32^ Briefly, exclusively breastfed term infants were randomly selected to receive 1.8 × 10^10^ colony-forming units (CFU) *B. infantis* EVC001 daily for 21 days (EVC001) starting at day 7 postnatal or to receive breast milk alone (control) and followed up to postnatal day 60.^32^ All mothers received lactation support throughout the study. The demographic information (e.g., age, sex, and gestational age) was collected from each participant. Here stool samples from randomly selected infants who were fed EVC001 (*n* = 20) and control infants (*n* = 20) on days 6 (Baseline), 40, and 60 postnatal were collected and analyzed for multiple proinflammatory cytokines using multiplexed immunoassays and levels of fecal calprotectin with an enzyme-linked immunosorbent assay (ELISA).

### Fecal microscopy

All samples were handled in an identical manner and analysis was completed by a blinded researcher. Samples were fixed by adding equal amount of 5% glutaraldehyde in 0.2 M cacodylate buffer (final concentration 2.5% glutaraldehyde in 0.1 M cacodylate buffer) for 1−2 h before being processed for Gram-stained light microscopy and scanning electron microscopy (SEM). For light microscopy, conventional Gram-stained samples on slides were imaged under an EVOS *Auto-FL* system using a ×60 lens and ×2.7 optical zoom, under the same setting of the color camera. Five random fields of images were collected from each sample slide. For SEM, since the samples were previously frozen before fixation, the osmium post-fixation and critical-point dry procedures were not performed. The fixed samples were dehydrated through an ethanol series and placed on membrane filters. The samples were mounted onto the SEM stubs, aired overnight, and then vacuum-oven dried at 50 °C for >2 h before sputter-coating with a thin layer of chromium using Denton Desk V sputter. Images were collected under various magnifications to capture bacterial morphology using a Hitachi S4700 field-emission SEM.

### Bacterial DNA methods

The relative abundance of the stool bacteria at the phylum, class, order, family, and genus level was characterized by performing a sequence analysis of the V4 segment of the 16S rRNA gene using QIIME v1.9.1 as previously described.^27^

### Fecal calprotectin

The level of fecal calprotectin was quantified using the IDK Calprotectin ELISA Kit (Immundiagnostik AG, Germany) in accordance with the manufacturer’s instructions. Absorbance was read at a wavelength of 450 nm using a Synergy HT Multi-Detection Microtiter Plate Reader (BioTek, Winooski, VT). The samples were plated in duplicate and the assay was performed twice.

### Multiplexed immunoassays

IL-1β, IL-2, IL-5, IL-6, IL-8, IL-10, IL-22, IFNγ, and TNFα were quantified from 80 mg of stool diluted 1:10 in Meso Scale Discovery (MSD; Rockville, MD) diluent using the U-PLEX Inflammation Panel 1 (human) Kit according to the manufacturer’s instructions as previously published.^33^ Standards and samples were measured in duplicate and blank values were subtracted from all readings. The plate was then read on Sector Imager 2400 MSD Discovery Workbench analysis software.

### Statistical analysis

Demographic differences between control and EVC001-fed infants were analyzed using Fisher’s Exact test for categorical data and Wilcoxon rank-sum (Mann-Whitney U) test for continuous data. Refer to Supplemental Table S1 for data eliminated from analysis or visualization. The relationship between fecal calprotectin concentration and %*Bifidobacteriaceae* was quantified using Spearman’s Rho correlation and the differences in calprotectin concentration between high (>25%) and low (<25%) *Bifidobacteriaceae* abundance was assessed using Wilcoxon rank-sum test. One subject with abnormally high fecal calprotectin concentration (>3 standard deviations above the mean of both treatment and control data) was considered an extreme outlier and removed from the aforementioned calprotectin analyses. Wilcoxon rank-sum tests were performed to assess relative abundance for each bacterial taxa and cytokine concentration differences. For radar plots, medians were adjusted to log scale, then normalized within each cytokine group from 0 to 1. Differences over time within each cytokine group were evaluated using Wilcoxon rank-sum test. *P* values were adjusted using the Bonferroni–Holms method and considered statistically significant if *P* < 0.05. The significance of differences in global cytokine profiles was determined by computing Bray–Curtis distance metrics translated into a principal coordinate analysis and visualized with EMPeror.^29^ Global cytokine profile differences by group status were then determined using a permutational multivariate analysis of variance, and significant *P* values were determined using 999 Monte Carlo permutations. To assess the relationship between the global cytokine profile and the microbiome, we used a Procrustes analysis.^34^ The taxonomic operational taxonomic unit table at the family level was computed using QIIME and the cytokine table was used to generate a distance matrix for each using a weighted UniFrac for 16S and Bray–Curtis for the cytokine. We performed a principal coordinate analysis separately on the two matrices and used a Procrustes analysis as implemented in QIIME to rotate, translate, and scale the matrices. The resulting transformed matrices were plotted using EMPeror.^34^ *P* values for the Procrustes analysis were generated using Monte Carlo simulations (*n* = 999). Raw correlation statistics were assigned the likelihood of these associations to be true positive associations by computing *P* values via a Fisher’s *Z* transform to normalize the distribution of the correlation scores.

## Results

### Demographics and fecal analyses of the infant participants

The characteristics of the study population included in the current analyses are presented in Table 1. Subjects in the EVC001 and control groups did not differ with respect to the parameters selected for randomization; however, the control infants were born to mothers who were significantly more likely to be first time mothers (*P* < 0.01) and significantly younger than the mothers whose infants were randomized to receive *B. infantis* EVC001 (*P* < 0.01). There were no significant differences between the groups with respect to hours in labor, birth mode, antibiotic use during labor, gestational age, sex, birth or discharge weight, birth length, maternal body mass index or gestational weight gain, or maternal group B Streptococcus diagnosis (Table 1).

**Table 1:** Participant characteristics of treatment groups in the inflammation cohort

	Controls	EVC001	*P* value
Treatment	20	20	
Home births	1	2	0.595
Hours in labor	18.11 (24.3)	10.94 (9.93)	0.574
Cesarean births	8	3	0.160
Antibiotics during labor	8	7	0.213
Gestation (weeks)	39.76 (1.13)	39.51 (1.13)	0.491
Females	11	11	0.752
Baby birth weight (g)	3555.74 (723.58)	3411.23 (338.65)	0.930
Baby discharged weight (g)	3324.65 (675.18)	3209.33 (379.47)	0.912
Baby birth length (cm)	50.54 (3.37)	50.31 (2.09)	0.724
Baby received antibiotics	1	0	1
Baby birth medical complications	3	0	0.232
Consumed infant formula before discharge	1	0	1
Maternal pre-pregnancy BMI calculated	23.538 (3.53)	25.610 (3.45)	0.059
Maternal pregnancy weight gain (kg)	15.33 (5.07)	13.940 (5.86)	0.66
Maternal GBS test positive	5	6	0.724
Primaparous	17	7	**0.006**
Maternal age	30.9 (3.42)	34.7 (3.97)	**0.004**

*BMI* body mass index, *GBS* group B Streptococcus

Statistically significant values are in bold *P* < 0.05

### Infants fed *B. infantis* EVC001 had significantly increased abundance of *Bifidobacteriaceae* in the infant gut microbiome

We first evaluated the microbiome profile from the two groups on day 6 (Baseline), day 40, and day 60 postnatal (Supplemental Table S2). The infants included in this subset exhibited similar microbiomes to those previously reported,^27^ in which no statistical differences between the two groups in the four major representative taxa (*Bifidobacteriaceae*, *Bacteroidaceae*, *Bifidobacteriaceae*, *Clostridiaceae*, and *Enterobacteriaceae*) were identified on day 6 postnatal, prior to the start of supplementation on day 7 (Supplemental Fig. S1a). Notably, at day 40, there was a significantly higher abundance of *Bifidobacteriaceae* in the *B. infantis* EVC001-fed group compared to controls (*P* < 0.0001; Supplemental Fig. S1a). Conversely, the abundances of *Bacteroidaceae* and *Clostridiaceae* were significantly lower, and *Enterobacteriaceae* was significantly lower in infants who were fed EVC001 (*P* < 0.05, *P* < 0.05, and *P* < 0.0001, respectively; Supplemental Fig. S1b). Similarly, on day 60 postnatal (32 days after last feed with EVC001), the microbiome composition of the infants fed EVC001 displayed higher abundance of *Bifidobacteriaceae*, as well as lower abundances of *Bacteroidaceae* and *Clostridiaceae* compared to the control infants (all *P* < 0.0001; Supplemental Fig. S1c).

**Fig. 1:**
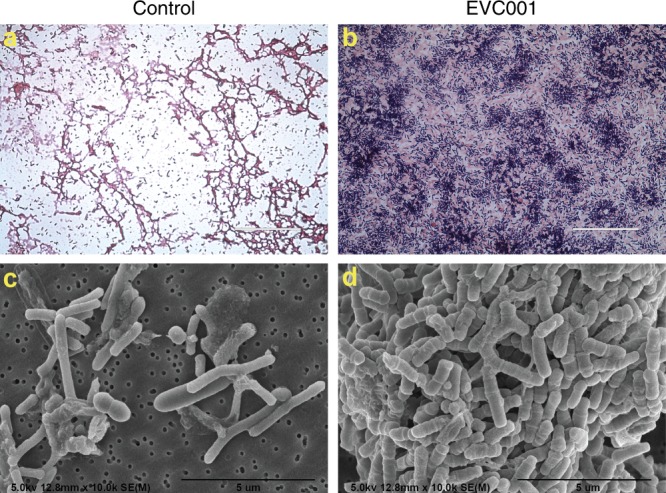
Microscopic analysis of the infant gut microbiome. Gram stain light microscopy (**a**, **b**) and scanning electron microscopy (**c**, **d**) micrographs of diluted fecal samples on day 40 postnatal from the control infants (**a**, **c**) and infants fed EVC001 (**b**, **d**). Scale bars: 50 µm (**a**, **b**) and 5 µm (**c**, **d**)

To further examine the marked changes in microbiome composition previously described,^27^ light and SEM were used to examine three fecal samples from day 40 from each of the control and EVC001-fed groups. Gram staining showed fecal smears of controls contained predominantly Gram-negative bacteria, while samples from EVC001-fed infants overwhelmingly contained Gram-positive bacteria (Fig. 1a, b). Multiple fields of view of the fecal samples from the control group identified several distinct bacterial morphologies, whereas samples from the EVC001-fed infants exhibited a uniform morphology of rod-shaped bacteria that are infrequently longitudinally split (Fig. 1c, d), which is in agreement with our molecular observations (Supplemental Fig. S1).

### Fecal calprotectin levels are directly correlated with the abundance of *Bifidobacteriaceae*

**Fig. 2:**
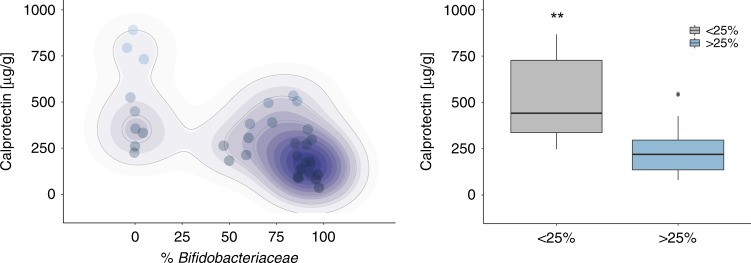
Fecal calprotectin levels are dependent on the abundance of *Bifidobacteriaceae*. Forty fecal samples from day 40 postnatal were evaluated for the concentration of fecal calprotectin and *Bifidobacteriaceae* abundance (****P < 0.0001; *r*_*s*_ = −0.72; **a**) and subdivided based on Bifidobacteriaceae abundance < or >25% (**b**). The data set is representative of at least three different experiments completed in duplicate and a non-parametric Wilcoxon rank-sum test was used to determine significance with the corresponding *P* values adjusted and considered statistically significant if *P < 0.05. **P < 0.01

Dysbiosis, including low abundance of *Bifidobacteriaceae* in the infant gut, has been associated with increased inflammation.^4,13^ Fecal samples from day 40 showed a significant correlation between *Bifidobacterium* abundance and lower fecal calprotectin levels (*r*_s_ = −0.72, *P* < 0.0001; Fig. 2a). These data also provide a clear bimodal distribution in which *Bifidobacteriaceae* abundance ≤25% is considered low *Bifidobacteriaceae* and >25% represents high *Bifidobacteriaceae* (Fig. 2a). Using a 25% abundance cut-off, samples that contained low *Bifidobacteriaceae* showed significantly increased enteric inflammation as measured by fecal calprotectin compared to samples that contained high levels of *Bifidobacteriaceae* (*P* < 0.01; Fig. 2b).

### Colonization with *B. infantis* EVC001 is associated with decreased fecal proinflammatory cytokine expression

**Fig. 3:**
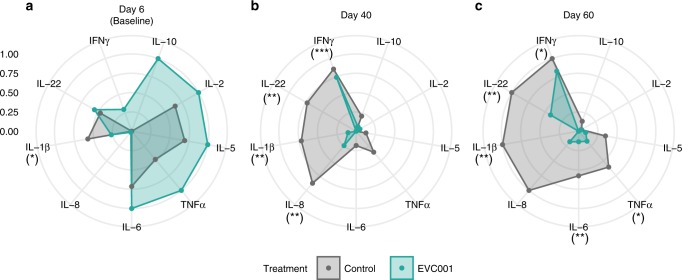
Fecal cytokine signature of infants who received *B. infantis* EVC001. Radar plot representations of median cytokine concentrations [pg/mg] detected in fecal samples from the controls (*n* = 20) and infants fed *Bifidobacterium infantis* EVC001 (*n* = 20; EVC001) on **a** day 6 (Baseline), **b** day 40 postnatal, and **c** day 60 postnatal. Median values were adjusted to log scale, then normalized within each cytokine group from 0 to 1. Statistical analysis was completed using Wilcoxon rank-sum test. *P* values were adjusted using Bonferonni–Holm method and considered statistically significant if *P < 0.05; **P < 0.01; ***P < 0.001

We next sought to determine whether colonization with *B. infantis* EVC001 could modulate intestinal cytokine production. At baseline, there was a significantly higher concentration of IL-1β production in the control compared with EVC001 infants (*P* < 0.05; Fig. 3a); however, no other statistically significant differences could be identified between the two groups.

By day 40 postnatal, we observed a significant modulation of the fecal cytokine profiles in the EVC001-fed infants, compared to the infants in the control group. Specifically, fecal concentrations of IL-8, IL-22, IL-1β, and IFNγ were significantly lower in EVC001 infants compared to the controls (Fig. 3b). At day 60, the controls produced significantly higher levels of IL-6, IL-22, TNFα, IL-1β, and IFNγ compared to infants who were colonized with *B. infantis* EVC001 (Fig. 3c). Taken together, these data show major global cytokine differences between exclusively breastfed infants fed EVC001 compared to matched exclusively breastfed control term infants over the first 60 days of life (Table 2).

**Table 2:** Significance of fecal cytokine signature of infants who received *B. infantis* EVC001

Cytokine	Day 6 (Baseline)			Day 40			Day 60		
	Median (SD), pg/mg			Median (SD), pg/mg			Median (SD), pg/mg		
	Control	EVC001	*P* value	Control	EVC001	*P* value	Control	EVC001	*P* value
IL-2	17.93 (1116.6)	48.13 (39.48)	1	2.87 (7.8)	3.36 (4.41)	1	3.23 (12.85)	3.16 (2.26)	1
IL-5	7.95 (138.82)	16.53 (10.87)	1	2.02 (2.1)	1.48 (1.3)	0.84	3.47 (7.06)	1.85 (1.63)	0.49
IL-6	3.98 (9.07)	9.29 (8.43)	1	0.83 (10.71)	0.48 (1)	0.63	2.65 (9.22)	0.71 (1.12)	**0.0054**
IL-8	46.49 (2023.91)	48.35 (406.67)	1	354.84 (4383.55)	81.67 (87.87)	**0.0054**	469.98 (3944.92)	70.13 (736.71)	0.069
IL-10	NA (NA)	8.76 (5.56)	NA	0.85 (2.26)	0.55 (0.4)	0.82	0.68 (0.6)	0.46 (0.76)	0.82
IL-22	3.77 (2.68)	4.22 (2.17)	1	5.29 (36.85)	2.07 (2.07)	**0.0023**	7.46 (17.35)	3.54 (3.43)	**0.0098**
TNFα	8.04 (154.19)	26.71 (20.47)	0.16	6.07 (67.16)	2.7 (2.62)	0.086	10.82 (14.67)	3.97 (2.67)	**0.012**
IL-1β	132.95 (1431.15)	38.65 (168.63)	**0.026**	237.02 (9241)	20.27 (74.56)	**0.0037**	724.61 (2773.7)	13.22 (193.24)	**0.0038**
IFNγ	0.02 (0.34)	0.29 (2.14)	0.1	34.26 (176.69)	12.99 (18.16)	**0.00073**	119.1 (380.22)	26.74 (43.16)	**0.013**

*IFN* interferon, *IL* interleukin, *NA* not applicable, *TNF* tumor necrosis factor

Statistically significant values are in bold *P* < 0.05

We sought to determine whether differences in fecal cytokines levels over time between control infants and EVC001-fed infants were influenced by postnatal age. These data are summarized in Supplemental Fig. S2 and Table 3. In general, fecal cytokine levels were significantly lower in infants who received EVC001 and remained low during the first 60 days postnatal, while the control infants had varying levels dependent on the cytokine, with several cytokine levels increasing over time.

**Table 3:** Significance of fecal cytokine concentration changes postnatally

Day 6 (Baseline) to Day 40						
Cytokine	Control			EVC001		
	Median (SD), pg/mg			Median (SD), pg/mg		
	Day 6 (Baseline)	Day 40	*P* value	Day 6 (Baseline)	Day 40	*P* value
IL-2	17.93 (1116.6)	2.87 (7.8)	**0.03**	48.13 (39.48)	3.36 (4.41)	**1.40E-05**
IL-5	7.95 (138.82)	2.02 (2.1)	**0.0084**	16.53 (10.87)	1.48 (1.3)	**1.30E-06**
IL-6	3.98 (9.07)	0.83 (10.71)	0.31	9.29 (8.43)	0.48 (1)	**1.50E-05**
IL-8	46.49 (2023.91)	354.84 (4383.55)	0.17	48.35 (406.67)	81.67 (87.87)	1
IL-10	NA (NA)	0.85 (2.26)	NA	8.76 (5.56)	0.55 (0.4)	**6.70E-06**
IL-22	3.77 (2.68)	5.29 (36.85)	0.17	4.22 (2.17)	2.07 (2.07)	**0.01**
TNFα	8.04 (154.19)	6.07 (67.16)	1	26.71 (20.47)	2.7 (2.62)	**1.80E-05**
IL-1β	132.95 (1431.15)	237.02 (9241)	1	38.65 (168.63)	20.27 (74.56)	0.58
IFNγ	0.02 (0.34)	34.26 (176.69)	**9.30E-06**	0.29 (2.14)	12.99 (18.16)	**0.0001**

Day 6 (Baseline) to Day 60						
	Control			EVC001		
	Median (SD), pg/mg			Median (SD), pg/mg		
Cytokine	Day 6 (Baseline)	Day 60	*P* value	Day 6 (Baseline)	Day 60	*P* value
IL-2	17.93 (1116.6)	3.23 (12.85)	0.4	48.13 (39.48)	3.16 (2.26)	**1.10E-05**
IL-5	7.95 (138.82)	3.47 (7.06)	0.21	16.53 (10.87)	1.85 (1.63)	**2.40E-06**
IL-6	3.98 (9.07)	2.65 (9.22)	0.89	9.29 (8.43)	0.71 (1.12)	**3.20E-06**
IL-8	46.49 (2023.91)	469.98 (3944.92)	0.17	48.35 (406.67)	70.13 (736.71)	1
IL-10	NA (NA)	0.68 (0.6)	NA	8.76 (5.56)	0.46 (0.76)	**6.30E-06**
IL-22	3.77 (2.68)	7.46 (17.35)	**0.0079**	4.22 (2.17)	3.54 (3.43)	0.37
TNFα	8.04 (154.19)	10.82 (14.67)	1	26.71 (20.47)	3.97 (2.67)	**6.90E-05**
IL-1β	132.95 (1431.15)	724.61 (2773.7)	1	38.65 (168.63)	13.22 (193.24)	0.058
IFNγ	0.02 (0.34)	119.1 (380.22)	**8.30E-06**	0.29 (2.14)	26.74 (43.16)	**5.00E-05**

Day 40 to Day 60						
	Control			EVC001		
	Median (SD), pg/mg			Median (SD), pg/mg		
Cytokine	Day 40	Day 60	*P* value	Day 40	Day 60	*P* value
IL-2	2.87 (7.8)	3.23 (12.85)	0.81	3.36 (4.41)	3.16 (2.26)	0.81
IL-5	2.02 (2.1)	3.47 (7.06)	0.21	1.48 (1.3)	1.85 (1.63)	0.33
IL-6	0.83 (10.71)	2.65 (9.22)	0.072	0.48 (1)	0.71 (1.12)	0.89
IL-8	354.84 (4383.55)	469.98 (3944.92)	1	81.67 (87.87)	70.13 (736.71)	1
IL-10	0.85 (2.26)	0.68 (0.6)	0.25	0.55 (0.4)	0.46 (0.76)	0.13
IL-22	5.29 (36.85)	7.46 (17.35)	0.27	2.07 (2.07)	3.54 (3.43)	**0.038**
TNFα	6.07 (67.16)	10.82 (14.67)	1	2.7 (2.62)	3.97 (2.67)	0.36
IL-1β	237.02 (9241)	724.61 (2773.7)	1	20.27 (74.56)	13.22 (193.24)	0.47
IFNγ	34.26 (176.69)	119.1 (380.22)	0.062	12.99 (18.16)	26.74 (43.16)	0.054

*IFN* interferon, *IL* interleukin, *NA* not applicable, *TNF* tumor necrosis factor

Statistically significant values are in bold *P* < 0.05

### Colonization with *B. infantis* EVC001 influences cytokine profiles

**Fig. 4:**
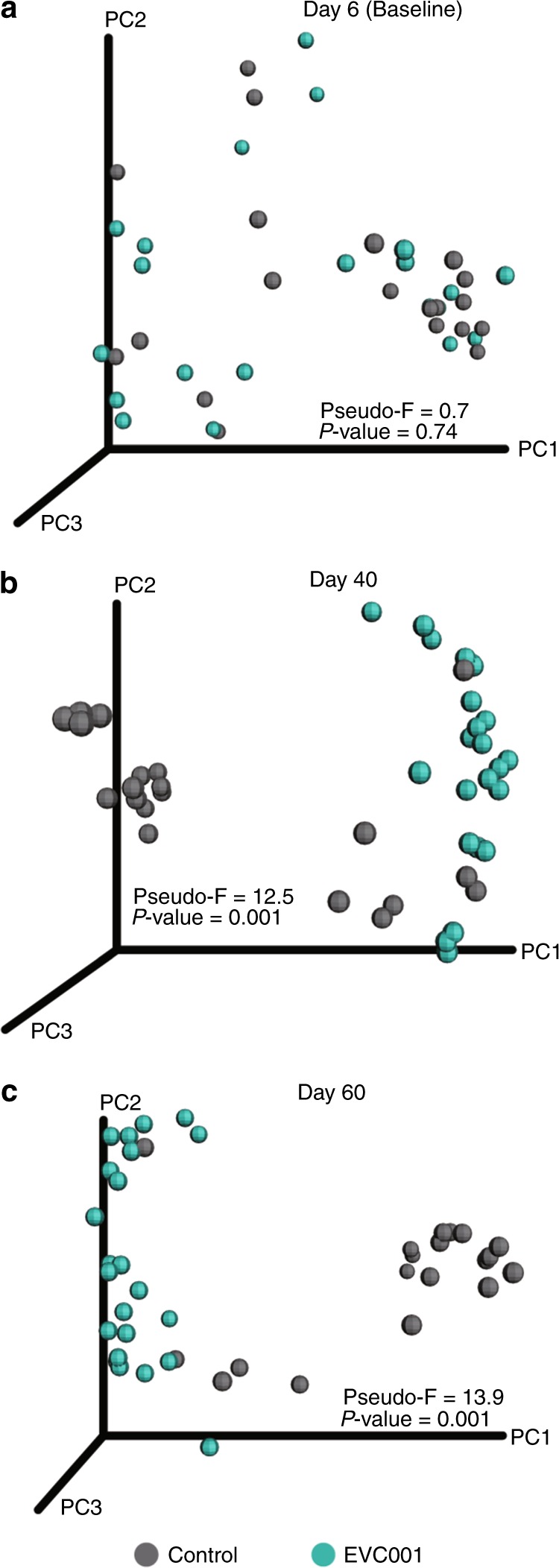
Principal coordinate analysis (PCoA) of global cytokine profiles according to group status. PCoA based on Bray–Curtis dissimilarity of global cytokine profiles between EVC001-fed infants and controls at **a** day 6 (Baseline), **b** day 40, and **c** day 60 postnatal

To identify the main driver of the measured fecal cytokines in our infant population, we used a principal component analysis as a dimension-reduction technique using all parameters of clinical data stated above, proinflammatory cytokine concentrations, and group. With the addition of the clinical data, the cytokine profile composition did not differ among the infants on day 6 (prior to receiving EVC001; Fig. 4a); however, by day 40, distinct clustering was evident between the EVC001-fed and control groups (*P* = 0.001, Pseudo-*F* = 12.5; Fig. 4b). Such separation remained evident on day 60, with more pronounced clustering compared to the earlier time points (*P* = 0.001, Pseudo-*F* = 13.9; Fig. 4c). Together, these observations suggest that time and colonization with *B. infantis* EVC001 are the primary factors influencing differences in fecal cytokines.

### Significant correlations exist between gut microbial abundance and intestinal inflammatory cytokine responses

**Fig. 5:**
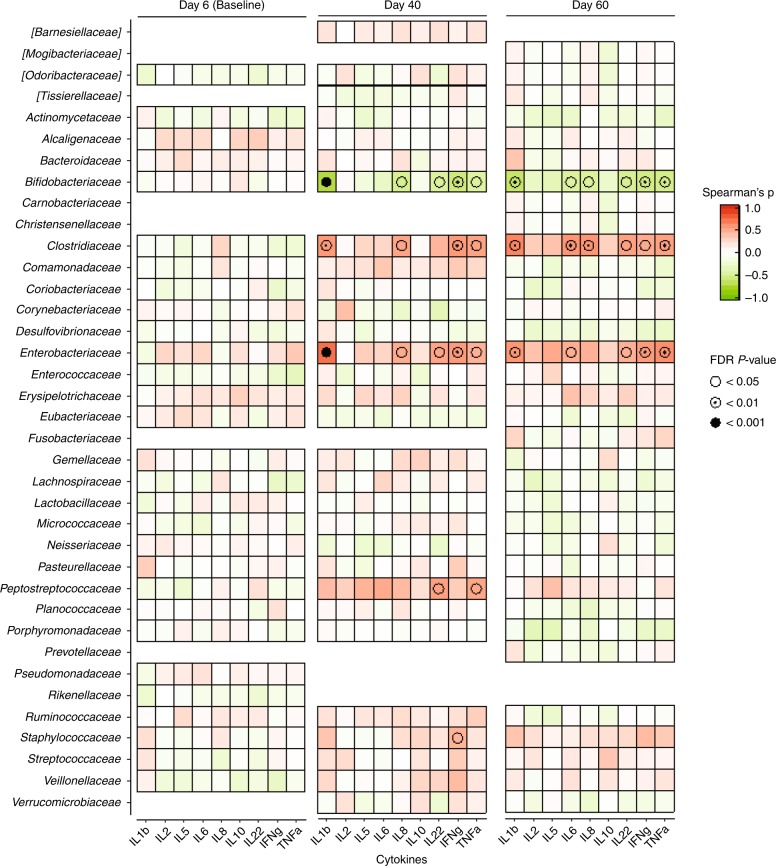
Correlations between specific gut taxa and intestinal inflammatory cytokine responses. Heatmap shows correlation between bacterial families and specific cytokines computed via Spearman correlation. *P* values are corrected using Benjamini–Hochberg procedure (false discovery rate) to estimate significant correlations between microbial taxonomic composition and specific cytokine concentration detected in the feces of exclusively breastfed infants at three time points (day 6 (Baseline), day 40, and day 60 postnatal). Each cytokine was tested in duplicate at three different time points. *P* values were adjusted and considered to be statistically significant if *P* < 0.05 (empty circle); *P* < 0.01 (semi-solid circle); *P* < 0.001 (solid circle)

We performed pairwise correlation tests between the microbial taxonomic composition and specific cytokine concentration detected in the feces of exclusively breastfed infants on day 6 (Baseline) as well as on days 40 and 60 (Spearman correlation with Benjamini–Hochberg false discovery rate correction *α* < 0.02). A total of four taxa were significantly correlated with specific proinflammatory cytokines, including, *Clostridiaceae*, *Enterobacteriaceae*, *Peptostreptococcaceae*, and *Staphylococcaceae*. Specifically, *Clostridiaceae* was significantly positively correlated with the production of IL-1β, IL-8, IFNγ, and TNFα at day 40 and IL-1β, IL-6, IL-8, IL-22, IFNγ, and TNFα at day 60. *Enterobacteriaceae* was significantly positively correlated with levels of IL-1β, IL-8, IL-22, IFNγ, and TNFα on day 40 and IL-1β, IL-6, IL-22, IFNγ, and TNFα at day 60 postnatal; *Peptostreptococcaceae* significantly positively correlated with IL-22 and TNFα on day 40; and *Staphylococcaceae* positively correlated with IFNγ concentration on day 40. Furthermore, five proinflammatory cytokines (IL-1β, IL-8, IL-22, IFNγ, and TNFα) were negatively correlated with *Bifidobacteriaceae* at day 40, as well as six proinflammatory cytokines (IL-1β, IL-6, IL-8, IL-22, IFNγ, and TNFα) negatively correlated at day 60 (Fig. 5; *P* values and Spearman’s rho available in Supplemental Table S3).

## Discussion

In this prospective study, we investigated the relationship between the gut microbiome and enteric inflammation in healthy, exclusively breastfed term infants.^32^ Specifically, we used fecal calprotectin, a well-characterized protein complex derived from neutrophils and macrophages in the mucosa,^13^ and fecal cytokine levels as markers of enteric inflammation to examine whether colonization with *B. infantis* EVC001 played a role in modulating intestinal inflammation. Our findings revealed a significant association between the bacterial phylogenetic structure at the family level in the infant fecal communities and the inflammatory status of their respective hosts for any day tested (*P* = 0, respectively; Supplemental Fig. S3). Furthermore, intestinal dysbiosis characterized by high levels of *Proteobacteria*^26^ and low levels of *Bifidobacteriaceae* strongly associated with higher levels of intestinal inflammation, as indicated by increased fecal inflammatory cytokine production (e.g., TNFα, IFN-γ, and IL-8) and calprotectin. Moreover, colonization with *B. infantis* EVC001 during the early stages of infancy significantly reduced inflammatory markers in the intestine along with previously demonstrated resolution of dysbiosis demonstrating that gut microbiome composition may directly modulate enteric inflammation.

Importantly, the infant intestinal microbiome is critical to the adequate development of mucosal and systemic immunity^2,3,7,35^; however, intestinal dysbiosis is now commonly observed in industrialized nations,^7,26,27^ which suggests that aberrations to the microbiome might impair the proper development of the immune system in infants today. Recent evidence has identified a “critical window” of immune development in which aberrations in the enteric microbial composition are most influential to immune system development and directly impact the health trajectory of the infant.^1–3^ Indeed, a growing body of scientific evidence has established that intestinal dysbiosis during infancy is associated with a number of autoimmune and allergic diseases later in life.^2,3,7,21^ Conversely, however, the presence of bifidobacterial species (e.g., *B. infantis*) is associated with decreased intestinal dysbiosis and a significantly decreased risk of autoimmune and allergic disease development,^7^ including inflammatory conditions such as eczema.^21^ Moreover, *Bifidobacteriaceae*, specifically *B. infantis*, colonization in infancy has been shown to improve the development of the host immune system, leading to enhanced CD4 T cell responses and increased IgA and IgG titers to vaccines in infancy and at 2 years.^28^ Importantly, these same reports showed that reduced vaccine response correlated with greater microbial diversity and increased *Enterobacteriaceae* abundance, which suggests that impaired immune development may be a consequence of gut dysbiosis and enteric inflammation as seen in control infants here, whereas the infants fed EVC001 exhibited decreased inflammation during this critical window of lymphocyte programming and immune system development.

Furthermore, our data showing infants with a gut microbiome comprised of high abundance of *Enterobacteriaceae* and *Clostridiaceae* had enteric inflammation are particularly intriguing since they provide further evidence that *Enterobacteriaceae* can manipulate immune activation to improve their own fitness and survival in the gut.^35^ This is important given the recently published link between gut dysbiosis, *Enterobacteriaceae*, and fecal calprotectin in infants suffering from colic^4^ and in preterm infants with a relative abundance of pathogenic *Klebsiella*.^12^ To this point, our data showed increased fecal calprotectin levels in infants without *Bifidobacteriaceae*, which has implications on lifelong health as shown in the findings by Orivuori et al.,^36^ in which intestinal inflammation early in life was predictive of an increased risk of developing autoimmune and allergic disease later in life,^36^ and a study linking increased fecal calprotectin to acute intestinal distress in preterm infants.^15^

Cytokines and chemokines are central to initiation of the immune response following pathogen exposure or tissue insult. Proinflammatory cytokines (e.g., TNFα, IL-1β, and IFNγ) are essential not only for mediating the inflammatory response but also individually and synergistically increasing intestinal permeability by disrupting enterocyte tight junctions leading to increased antigenic (e.g., lipopolysaccharide) penetration into the underlying lamina propria and further activation of the inflammatory response. This combination of local inflammation and increased intestinal permeability has been linked to several pathological conditions (reviewed in ref. ^24^). The observed differences in fecal calprotectin and several fecal cytokines between exclusively breastfed control infants and those fed *B. infantis* EVC001 provide insight into a potential mechanism of action by which chronic low-grade enteric inflammation in the former leads to dysbiosis-associated disease while the relative immune homeostasis in the latter is protective.

The role of *B. infantis* in reducing enteric inflammation has not been fully elucidated; however, previously published observations have described the importance of *B. infantis* colonization to improve binding affinity and reduce inflammation in intestinal epithelial cells in vitro.^37^ In addition, in animal models of NEC, *B. infantis* has been shown to decrease NEC and modulate dysbiosis-induced IL-6, TNFα, and inducible nitric oxide synthase production, as well as antimicrobial peptides (e.g., Reg3b and Reg3g).^36^ This is further supported in preclinical studies, in which the incidence of NEC was reduced in animals fed *B. infantis*. Specifically, the protective effect of *B. infantis* against the development of NEC has been attributed to decreased Toll-like receptor 2 (TLR2) and TLR4 expression, reduced intestinal inflammation as shown by reduction of TNFα and IL-8 production, and increased intestinal barrier function.^38^ Our data extend our current understanding to show specific *B. infantis-*induced reduction of enteric inflammation in healthy term infants in vivo, which suggest a critically important role for *B. infantis* in reducing and possibly preventing immune activation in the gut.

Although the precise mechanism modulating enteric inflammation remains unclear and a limitation of this study, the significantly lower levels of fecal endotoxin, reduced colonic glycan degradation, and significantly fewer mucolytic strains of bacteria (e.g., *Bacteroides*) in infants who had a microbiome dominated by *B. infantis* EVC001^27,31^ may explain the observed significant decrease in enteric inflammation. Moreover, *B. infantis* metabolites have been shown to modulate IL-1β-induced nuclear factor (NF)-κB p65 translocation in human fetal enterocytes,^39^ which is critical for the initiation of the inflammatory response. Indeed, historical publications indicate that *Bifidobacterium* spp. produce tryptophan metabolites,^40^ and more contemporary reports have identified indole-3-lactic acid as a *B. infantis*-specific metabolite that prevents intestinal inflammation in vitro through the activation of the aryl hydrocarbon receptor and NF erythroid 2-related factor (Nrf2)^41^; *Experimental Biology Meeting*). Taken together, these data indicate *B. infantis-*derived metabolites may directly modulate intestinal inflammation. Therefore, it is critical for future studies to elucidate the specific role of these metabolites in inducing immune homeostasis.

In conclusion, the present investigation extends our current understanding of how the composition of the gut microbiome directly modulates enteric inflammation and, for the first time, identifies intestinal inflammation in healthy exclusively breastfed infants. Furthermore, infants fed *B. infantis* EVC001 showed significantly decreased intestinal inflammation compared to those without *B. infantis*. These findings are critically important given recent studies linking gut dysbiosis to intestinal inflammation and an increased risk of developing colic, autoimmune, and allergic diseases.^2,4^ Together, these data provide a novel application by which the infant microbiome can be restored to prevent the induction of aberrant immune responses during a critically important window of immunological development.

## Supplementary information

Supplemental Figure S1

Supplemental Figure S2

Supplemental Figure S3

Supplemental Tables

Supplementary Figure Legends

## Data Availability

We are committed to making our data, materials, and analysis methods open and available upon request, where permitted.
