# Supplementary figures and images for: Colonization by B. infantis EVC001 modulates enteric inflammation in exclusively breastfed infants
Source: Pediatr Res. 2019 Aug 23;86(6):749–57. doi: 10.1038/s41390-019-0533-2 (PMC6887859; doi:10.1038/s41390-019-0533-2)

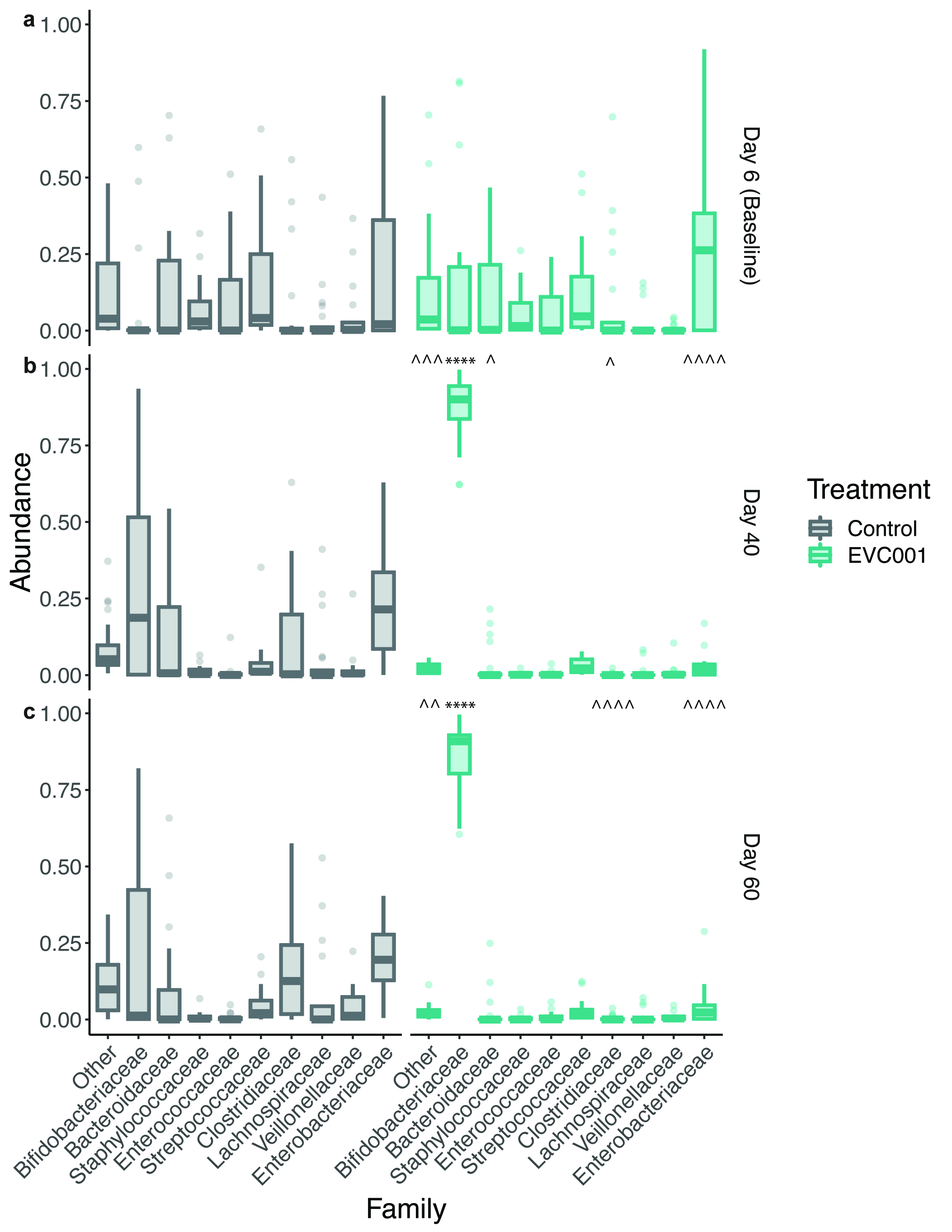

Supplement: Supplementary file 1 — Supplemental Figure S1 [file 41390_2019_533_MOESM1_ESM.tif]

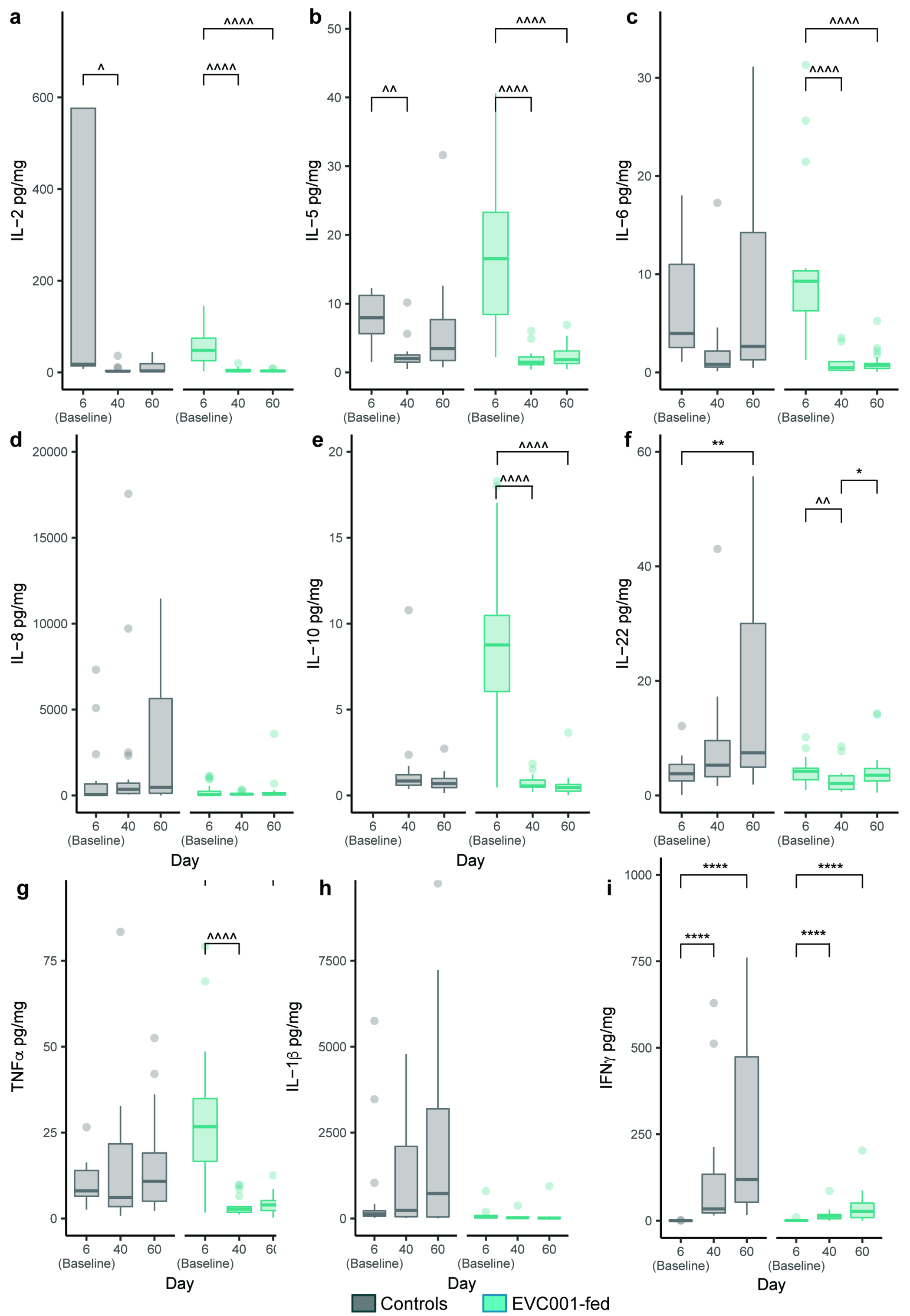

Supplement: Supplementary file 2 — Supplemental Figure S2 [file 41390_2019_533_MOESM2_ESM.tif]

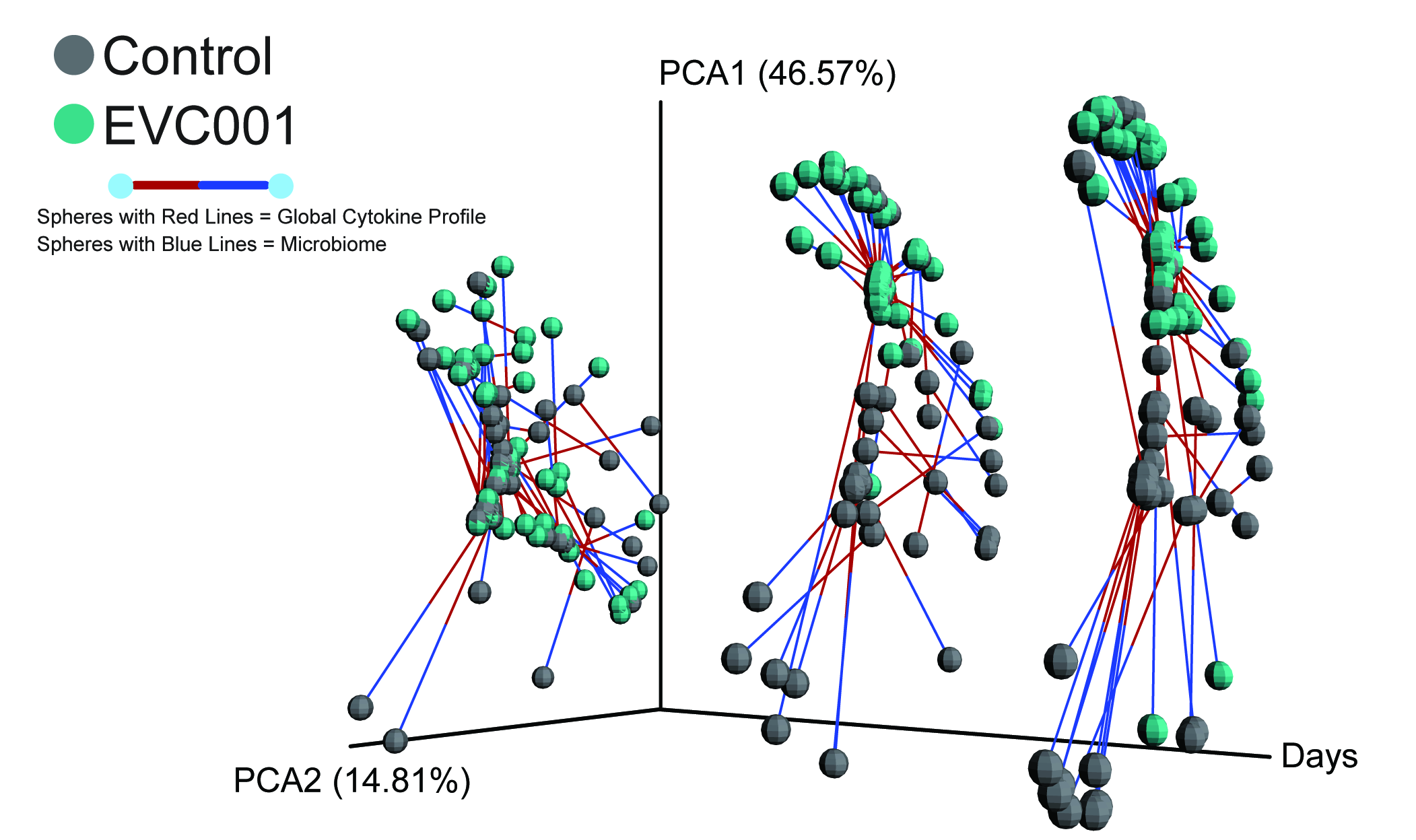

Supplement: Supplementary file 3 — Supplemental Figure S3 [file 41390_2019_533_MOESM3_ESM.tif]
