# Supplementary material for: Colonization by B. infantis EVC001 modulates enteric inflammation in exclusively breastfed infants
Source: Pediatr Res. 2019 Aug 23;86(6):749–57. doi: 10.1038/s41390-019-0533-2 (PMC6887859; doi:10.1038/s41390-019-0533-2)
Supplement: Supplementary file 4 — Supplemental Tables [file 41390_2019_533_MOESM4_ESM.docx]

| **Supplemental Table S1 Data eliminated from analysis or visualization** | | | | | |
| --- | --- | --- | --- | --- | --- |
| **Day** | **Treatment** | **Cytokine** | **Concentration** | **Data Action** | **Reason** |
| 6 (Baseline) | Control | IL2 | 2247.74 | not shown in boxplot | plot scale reduced to 0 - 750 to better showcase data |
| 6 (Baseline) | Control | IL5 | 399.91 | not shown in boxplot | plot scale reduced to 0 - 50 to better showcase data |
| 40 | Control | IL6 | 45.66 | not shown in boxplot | plot scale reduced to 0 - 35 to better showcase data |
| 40 | Control | IL8 | 43779.38 | excluded from analysis | extreme outlier (possible assay error) |
| 6 (Baseline) | Control | IL10 | 236.29 | excluded from analysis | extreme outlier (possible assay error) |
| 40 | Control | IL22 | 167.74 | not shown in boxplot | plot scale reduced to 0 - 60 to better showcase data |
| 6 (Baseline) | Control | TNFα | 606.48 | not shown in boxplot | plot scale reduced to 0 - 100 to better showcase data |
| 40 | Control | TNFα | 301.57 | not shown in boxplot | plot scale reduced to 0 - 100 to better showcase data |
| 40 | Control | IL-1β | 23899.20 | not shown in boxplot | plot scale reduced to 0 - 10000 to better showcase data |
| 40 | Control | IL-1β | 35845.38 | not shown in boxplot | plot scale reduced to 0 - 10000 to better showcase data |
| 60 | Control | IFNγ | 1476.40 | not shown in boxplot | plot scale reduced to 0 - 1000 to better showcase data |

| **Supplemental Table S2 Microbiome profile of infants that received *B. infantis* EVC001** | | | | | | | | | |
| --- | --- | --- | --- | --- | --- | --- | --- | --- | --- |
|  | **Day 6 (Baseline)** | | | **Day 40** | | | **Day 60** | | |
|  | **% Relative Abundance** | |  | **% Relative Abundance** | |  | **% Relative Abundance** | |  |
| **Family** | **Control** | **EVC001** | ***P* value** | **Control** | **EVC001** | ***P* value** | **Control** | **EVC001** | ***P* value** |
| **Bifidobacteriaceae** | 0.06978779 | 0.14718029 | 0.686 | 0.29389305 | 0.87120167 | **1.20E-06** | 0.22415323 | 0.85322628 | **1.10E-06** |
| **Bacteroidaceae** | 0.12747777 | 0.10404602 | 0.499 | 0.12973584 | 0.02752045 | **0.02331** | 0.10109035 | 0.02011562 | 0.0716 |
| **Staphylococcaceae** | 0.07224006 | 0.05432678 | 0.54 | 0.01300446 | 0.00364467 | 0.1956 | 0.01003416 | 0.00388429 | **0.0118** |
| **Enterococcaceae** | 0.09621277 | 0.05395919 | 0.884 | 0.00785173 | 0.0044948 | 0.54546 | 0.00663853 | 0.00818023 | 0.9019 |
| **Streptococcaceae** | 0.1534639 | 0.11689596 | 0.686 | 0.03856906 | 0.02907406 | 0.56182 | 0.04818214 | 0.02770279 | 0.2952 |
| **Clostridiaceae** | 0.072283 | 0.08737957 | 0.424 | 0.11413123 | 0.00156459 | **0.02259** | 0.16350622 | 0.00402528 | **3.60E-05** |
| **Lachnospiraceae** | 0.04028768 | 0.01971982 | 0.966 | 0.05032884 | 0.00722515 | 0.10413 | 0.08108918 | 0.00810084 | 0.1889 |
| **Veillonellaceae** | 0.04668136 | 0.00648194 | **0.033** | 0.02194156 | 0.0076837 | 0.1235 | 0.04530777 | 0.00763965 | 0.892 |
| **Enterobacteriaceae** | 0.19294682 | 0.27427214 | 0.593 | 0.23931813 | 0.02604114 | **9.60E-06** | 0.19401204 | 0.04269521 | **7.60E-05** |
| **Other** | 0.12861888 | 0.13573828 | 0.969 | 0.09122617 | 0.02154982 | **0.00048** | 0.12598644 | 0.02442983 | **0.0024** |

| **Supplemental Table S3 Correlations between gut microbial  abundance and intestinal inflammatory cytokine responses** | | | | |
| --- | --- | --- | --- | --- |
| **Cytokine** | **Day** | **Bacterial family** | **Spearman's ρ** | **FDR-P** |
| IFNg | Day 6 | Actinomycetaceae | -0.279277043 | 0.843924414 |
| IFNg | Day 6 | Aerococcaceae |  | 1 |
| IFNg | Day 6 | Aeromonadaceae |  | 1 |
| IFNg | Day 6 | Alcaligenaceae | 0.124469985 | 1 |
| IFNg | Day 6 | Bacillaceae |  | 1 |
| IFNg | Day 6 | Bacteroidaceae | 0.043304842 | 1 |
| IFNg | Day 6 | Barnesiellaceae |  | 1 |
| IFNg | Day 6 | Bifidobacteriaceae | -0.021481028 | 1 |
| IFNg | Day 6 | BS11 |  | 1 |
| IFNg | Day 6 | Burkholderiaceae |  | 1 |
| IFNg | Day 6 | Campylobacteraceae |  | 1 |
| IFNg | Day 6 | Carnobacteriaceae |  | 1 |
| IFNg | Day 6 | Christensenellaceae |  | 1 |
| IFNg | Day 6 | Clostridiaceae | -0.231039246 | 1 |
| IFNg | Day 6 | Comamonadaceae | 0.010291616 | 1 |
| IFNg | Day 6 | Coriobacteriaceae | -0.286293861 | 0.843924414 |
| IFNg | Day 6 | Corynebacteriaceae | 0.101969545 | 1 |
| IFNg | Day 6 | Dermabacteraceae |  | 1 |
| IFNg | Day 6 | Desulfovibrionaceae | -0.205927355 | 1 |
| IFNg | Day 6 | Dietziaceae |  | 1 |
| IFNg | Day 6 | Enterobacteriaceae | 0.205733518 | 1 |
| IFNg | Day 6 | Enterococcaceae | -0.275802148 | 0.843924414 |
| IFNg | Day 6 | Erysipelotrichaceae | 0.138376669 | 1 |
| IFNg | Day 6 | Eubacteriaceae | 0.120561972 | 1 |
| IFNg | Day 6 | Fibrobacteraceae |  | 1 |
| IFNg | Day 6 | Flavobacteriaceae |  | 1 |
| IFNg | Day 6 | Fusobacteriaceae |  | 1 |
| IFNg | Day 6 | Gemellaceae | 0.136270199 | 1 |
| IFNg | Day 6 | Helicobacteraceae |  | 1 |
| IFNg | Day 6 | Lachnospiraceae | -0.312118819 | 0.843924414 |
| IFNg | Day 6 | Lactobacillaceae | 0.104525885 | 1 |
| IFNg | Day 6 | Leptotrichiaceae |  | 1 |
| IFNg | Day 6 | Leuconostocaceae |  | 1 |
| IFNg | Day 6 | Micrococcaceae | 0.0450072 | 1 |
| IFNg | Day 6 | mitochondria |  | 1 |
| IFNg | Day 6 | Mogibacteriaceae |  | 1 |
| IFNg | Day 6 | Moraxellaceae |  | 1 |
| IFNg | Day 6 | Neisseriaceae | 0.015335184 | 1 |

| **Cytokine** | **Day** | **Bacterial family** | **Spearman's ρ** | **FDR-P** |
| --- | --- | --- | --- | --- |
| IFNg | Day 6 | Odoribacteraceae | -0.133957747 | 1 |
| IFNg | Day 6 | Other | -0.292166993 | 0.843924414 |
| IFNg | Day 6 | Other | -0.060725985 | 1 |
| IFNg | Day 6 | Other | -0.058517767 | 1 |
| IFNg | Day 6 | Other |  | 1 |
| IFNg | Day 6 | Other |  | 1 |
| IFNg | Day 6 | Paenibacillaceae |  | 1 |
| IFNg | Day 6 | Paraprevotellaceae |  | 1 |
| IFNg | Day 6 | Pasteurellaceae | 0.098995548 | 1 |
| IFNg | Day 6 | Peptococcaceae |  | 1 |
| IFNg | Day 6 | Peptostreptococcaceae | -0.139404621 | 1 |
| IFNg | Day 6 | Planococcaceae | 0.20739514 | 1 |
| IFNg | Day 6 | Porphyromonadaceae | -0.159591671 | 1 |
| IFNg | Day 6 | Prevotellaceae |  | 1 |
| IFNg | Day 6 | Pseudomonadaceae | 0.080374648 | 1 |
| IFNg | Day 6 | RFP12 |  | 1 |
| IFNg | Day 6 | Rikenellaceae | -0.133957747 | 1 |
| IFNg | Day 6 | Ruminococcaceae | -0.076047236 | 1 |
| IFNg | Day 6 | Spirochaetaceae |  | 1 |
| IFNg | Day 6 | Staphylococcaceae | 0.07876377 | 1 |
| IFNg | Day 6 | Streptococcaceae | 0.0710795 | 1 |
| IFNg | Day 6 | Succinivibrionaceae |  | 1 |
| IFNg | Day 6 | Tissierellaceae |  | 1 |
| IFNg | Day 6 | Turicibacteraceae |  | 1 |
| IFNg | Day 6 | Veillonellaceae | -0.315659923 | 0.843924414 |
| IFNg | Day 6 | Verrucomicrobiaceae |  | 1 |
| IFNg | Day 6 | Xanthomonadaceae |  | 1 |
| IFNg | Day 40 | Actinomycetaceae | 0.052652185 | 1 |
| IFNg | Day 40 | Aerococcaceae |  | 1 |
| IFNg | Day 40 | Aeromonadaceae |  | 1 |
| IFNg | Day 40 | Alcaligenaceae | 0.111306249 | 1 |
| IFNg | Day 40 | Bacillaceae |  | 1 |
| IFNg | Day 40 | Bacteroidaceae | 0.09794932 | 1 |
| IFNg | Day 40 | Barnesiellaceae | 0.111932422 | 1 |
| IFNg | Day 40 | Bifidobacteriaceae | -0.596254075 | 0.001007677 |
| IFNg | Day 40 | BS11 |  | 1 |
| IFNg | Day 40 | Burkholderiaceae |  | 1 |
| IFNg | Day 40 | Campylobacteraceae |  | 1 |
| IFNg | Day 40 | Carnobacteriaceae |  | 1 |
| IFNg | Day 40 | Christensenellaceae |  | 1 |
| IFNg | Day 40 | Clostridiaceae | 0.558340628 | 0.002015412 |
| IFNg | Day 40 | Comamonadaceae | 0.330160285 | 0.302903173 |
| IFNg | Day 40 | Coriobacteriaceae | -0.006978789 | 1 |
| IFNg | Day 40 | Corynebacteriaceae | -0.092380295 | 1 |
| IFNg | Day 40 | Dermabacteraceae |  | 1 |
| IFNg | Day 40 | Desulfovibrionaceae | -0.175536125 | 1 |
| IFNg | Day 40 | Dietziaceae |  | 1 |
| IFNg | Day 40 | Enterobacteriaceae | 0.552760784 | 0.002015412 |
| IFNg | Day 40 | Enterococcaceae | -0.008476516 | 1 |
| IFNg | Day 40 | Erysipelotrichaceae | -0.058032862 | 1 |
| IFNg | Day 40 | Eubacteriaceae | -0.161680165 | 1 |
| IFNg | Day 40 | Fibrobacteraceae |  | 1 |
| IFNg | Day 40 | Flavobacteriaceae |  | 1 |
| IFNg | Day 40 | Fusobacteriaceae |  | 1 |
| IFNg | Day 40 | Gemellaceae | 0.216903778 | 0.867908446 |
| IFNg | Day 40 | Helicobacteraceae |  | 1 |
| IFNg | Day 40 | Lachnospiraceae | 0.002379526 | 1 |
| IFNg | Day 40 | Lactobacillaceae | -0.052701806 | 1 |
| IFNg | Day 40 | Leptotrichiaceae |  | 1 |
| IFNg | Day 40 | Leuconostocaceae |  | 1 |
| IFNg | Day 40 | Micrococcaceae | 0.170085928 | 1 |
| IFNg | Day 40 | mitochondria |  | 1 |
| IFNg | Day 40 | Mogibacteriaceae |  | 1 |
| IFNg | Day 40 | Moraxellaceae |  | 1 |
| IFNg | Day 40 | Neisseriaceae | 0.024004768 | 1 |
| IFNg | Day 40 | Odoribacteraceae | 0.198990973 | 0.867908446 |
| IFNg | Day 40 | Other | 0.489381068 | 0.012980853 |
| IFNg | Day 40 | Other | -0.198990973 | 0.867908446 |
| IFNg | Day 40 | Other | 0.198990973 | 0.867908446 |
| IFNg | Day 40 | Other | 0.034708524 | 1 |
| IFNg | Day 40 | Other | 0.180841654 | 1 |
| IFNg | Day 40 | Paenibacillaceae |  | 1 |
| IFNg | Day 40 | Paraprevotellaceae |  | 1 |
| IFNg | Day 40 | Pasteurellaceae | 0.313434204 | 0.302903173 |
| IFNg | Day 40 | Peptococcaceae |  | 1 |
| IFNg | Day 40 | Peptostreptococcaceae | 0.314615637 | 0.302903173 |
| IFNg | Day 40 | Planococcaceae | 0.081947311 | 1 |
| IFNg | Day 40 | Porphyromonadaceae | 0.005538559 | 1 |
| IFNg | Day 40 | Prevotellaceae |  | 1 |
| IFNg | Day 40 | Pseudomonadaceae |  | 1 |
| IFNg | Day 40 | RFP12 |  | 1 |
| IFNg | Day 40 | Rikenellaceae |  | 1 |
| IFNg | Day 40 | Ruminococcaceae | 0.212835598 | 0.867908446 |
| IFNg | Day 40 | Spirochaetaceae |  | 1 |
| IFNg | Day 40 | Staphylococcaceae | 0.443223033 | 0.037895179 |
| IFNg | Day 40 | Streptococcaceae | 0.311607893 | 0.302903173 |
| IFNg | Day 40 | Succinivibrionaceae |  | 1 |
| IFNg | Day 40 | Tissierellaceae | 0.148984639 | 1 |
| IFNg | Day 40 | Turicibacteraceae |  | 1 |
| IFNg | Day 40 | Veillonellaceae | 0.400439678 | 0.088853146 |
| IFNg | Day 40 | Verrucomicrobiaceae | 0.198990973 | 0.867908446 |
| IFNg | Day 40 | Xanthomonadaceae |  | 1 |
| IFNg | Day 60C | Actinomycetaceae | -0.13347904 | 1 |
| IFNg | Day 60C | Aerococcaceae |  | 1 |
| IFNg | Day 60C | Aeromonadaceae |  | 1 |
| IFNg | Day 60C | Alcaligenaceae | -0.094003789 | 1 |
| IFNg | Day 60C | Bacillaceae |  | 1 |
| IFNg | Day 60C | Bacteroidaceae | 0.140745068 | 1 |
| IFNg | Day 60C | Barnesiellaceae |  | 1 |
| IFNg | Day 60C | Bifidobacteriaceae | -0.553786314 | 0.003807789 |
| IFNg | Day 60C | BS11 |  | 1 |
| IFNg | Day 60C | Burkholderiaceae |  | 1 |
| IFNg | Day 60C | Campylobacteraceae |  | 1 |
| IFNg | Day 60C | Carnobacteriaceae | 0.057983263 | 1 |
| IFNg | Day 60C | Christensenellaceae | 0.057983263 | 1 |
| IFNg | Day 60C | Clostridiaceae | 0.47387171 | 0.031576538 |
| IFNg | Day 60C | Comamonadaceae | -0.137885376 | 1 |
| IFNg | Day 60C | Coriobacteriaceae | -0.254842895 | 0.840812388 |
| IFNg | Day 60C | Corynebacteriaceae | 0.028186237 | 1 |
| IFNg | Day 60C | Dermabacteraceae |  | 1 |
| IFNg | Day 60C | Desulfovibrionaceae | -0.282674611 | 0.725495757 |
| IFNg | Day 60C | Dietziaceae |  | 1 |
| IFNg | Day 60C | Enterobacteriaceae | 0.552065214 | 0.003807789 |
| IFNg | Day 60C | Enterococcaceae | 0.069010574 | 1 |
| IFNg | Day 60C | Erysipelotrichaceae | 0.11461988 | 1 |
| IFNg | Day 60C | Eubacteriaceae | 0.083753602 | 1 |
| IFNg | Day 60C | Fibrobacteraceae |  | 1 |
| IFNg | Day 60C | Flavobacteriaceae |  | 1 |
| IFNg | Day 60C | Fusobacteriaceae | 0.173949788 | 1 |
| IFNg | Day 60C | Gemellaceae | -0.131919908 | 1 |
| IFNg | Day 60C | Helicobacteraceae |  | 1 |
| IFNg | Day 60C | Lachnospiraceae | -0.257283977 | 0.840812388 |
| IFNg | Day 60C | Lactobacillaceae | -0.096913077 | 1 |
| IFNg | Day 60C | Leptotrichiaceae |  | 1 |
| IFNg | Day 60C | Leuconostocaceae |  | 1 |
| IFNg | Day 60C | Micrococcaceae | -0.06588657 | 1 |
| IFNg | Day 60C | mitochondria |  | 1 |
| IFNg | Day 60C | Mogibacteriaceae | 0.057983263 | 1 |
| IFNg | Day 60C | Moraxellaceae |  | 1 |
| IFNg | Day 60C | Neisseriaceae | -0.032212924 | 1 |
| IFNg | Day 60C | Odoribacteraceae | 0.057983263 | 1 |
| IFNg | Day 60C | Other | 0.37816807 | 0.189139058 |
| IFNg | Day 60C | Other | -0.144257894 | 1 |
| IFNg | Day 60C | Other | 0.076211426 | 1 |
| IFNg | Day 60C | Other |  | 1 |
| IFNg | Day 60C | Other |  | 1 |
| IFNg | Day 60C | Paenibacillaceae |  | 1 |
| IFNg | Day 60C | Paraprevotellaceae |  | 1 |
| IFNg | Day 60C | Pasteurellaceae | 0.075294164 | 1 |
| IFNg | Day 60C | Peptococcaceae |  | 1 |
| IFNg | Day 60C | Peptostreptococcaceae | -0.045339489 | 1 |
| IFNg | Day 60C | Planococcaceae | -0.029033982 | 1 |
| IFNg | Day 60C | Porphyromonadaceae | -0.312071676 | 0.532735936 |
| IFNg | Day 60C | Prevotellaceae | 0.044458284 | 1 |
| IFNg | Day 60C | Pseudomonadaceae |  | 1 |
| IFNg | Day 60C | RFP12 |  | 1 |
| IFNg | Day 60C | Rikenellaceae |  | 1 |
| IFNg | Day 60C | Ruminococcaceae | -0.012758357 | 1 |
| IFNg | Day 60C | Spirochaetaceae |  | 1 |
| IFNg | Day 60C | Staphylococcaceae | 0.400569527 | 0.146931386 |
| IFNg | Day 60C | Streptococcaceae | 0.094971841 | 1 |
| IFNg | Day 60C | Succinivibrionaceae |  | 1 |
| IFNg | Day 60C | Tissierellaceae | 0.050963061 | 1 |
| IFNg | Day 60C | Turicibacteraceae |  | 1 |
| IFNg | Day 60C | Veillonellaceae | 0.101333556 | 1 |
| IFNg | Day 60C | Verrucomicrobiaceae | -0.167507203 | 1 |
| IFNg | Day 60C | Xanthomonadaceae |  | 1 |
| IL10 | Day 6 | Actinomycetaceae | -0.189732897 | 1 |
| IL10 | Day 6 | Aerococcaceae |  | 1 |
| IL10 | Day 6 | Aeromonadaceae |  | 1 |
| IL10 | Day 6 | Alcaligenaceae | 0.282195328 | 1 |
| IL10 | Day 6 | Bacillaceae |  | 1 |
| IL10 | Day 6 | Bacteroidaceae | 0.129724988 | 1 |
| IL10 | Day 6 | Barnesiellaceae |  | 1 |
| IL10 | Day 6 | Bifidobacteriaceae | 0.158173065 | 1 |
| IL10 | Day 6 | BS11 |  | 1 |
| IL10 | Day 6 | Burkholderiaceae |  | 1 |
| IL10 | Day 6 | Campylobacteraceae |  | 1 |
| IL10 | Day 6 | Carnobacteriaceae |  | 1 |
| IL10 | Day 6 | Christensenellaceae |  | 1 |
| IL10 | Day 6 | Clostridiaceae | -0.110985271 | 1 |
| IL10 | Day 6 | Comamonadaceae | -0.060159211 | 1 |
| IL10 | Day 6 | Coriobacteriaceae | -0.037496456 | 1 |
| IL10 | Day 6 | Corynebacteriaceae | 0.023727118 | 1 |
| IL10 | Day 6 | Dermabacteraceae |  | 1 |
| IL10 | Day 6 | Desulfovibrionaceae | 0.081796118 | 1 |
| IL10 | Day 6 | Dietziaceae |  | 1 |
| IL10 | Day 6 | Enterobacteriaceae | 0.205958035 | 1 |
| IL10 | Day 6 | Enterococcaceae | -0.147476829 | 1 |
| IL10 | Day 6 | Erysipelotrichaceae | 0.295116314 | 1 |
| IL10 | Day 6 | Eubacteriaceae | 0.161963027 | 1 |
| IL10 | Day 6 | Fibrobacteraceae |  | 1 |
| IL10 | Day 6 | Flavobacteriaceae |  | 1 |
| IL10 | Day 6 | Fusobacteriaceae |  | 1 |
| IL10 | Day 6 | Gemellaceae | -0.002235535 | 1 |
| IL10 | Day 6 | Helicobacteraceae |  | 1 |
| IL10 | Day 6 | Lachnospiraceae | -0.04785982 | 1 |
| IL10 | Day 6 | Lactobacillaceae | 0.147982289 | 1 |
| IL10 | Day 6 | Leptotrichiaceae |  | 1 |
| IL10 | Day 6 | Leuconostocaceae |  | 1 |
| IL10 | Day 6 | Micrococcaceae | -0.139224237 | 1 |
| IL10 | Day 6 | mitochondria |  | 1 |
| IL10 | Day 6 | Mogibacteriaceae |  | 1 |
| IL10 | Day 6 | Moraxellaceae |  | 1 |
| IL10 | Day 6 | Neisseriaceae | 0.093461424 | 1 |
| IL10 | Day 6 | Odoribacteraceae | -0.132515204 | 1 |
| IL10 | Day 6 | Other | -0.285070973 | 1 |
| IL10 | Day 6 | Other | -0.127021032 | 1 |
| IL10 | Day 6 | Other | 0.057644895 | 1 |
| IL10 | Day 6 | Other |  | 1 |
| IL10 | Day 6 | Other |  | 1 |
| IL10 | Day 6 | Paenibacillaceae |  | 1 |
| IL10 | Day 6 | Paraprevotellaceae |  | 1 |
| IL10 | Day 6 | Pasteurellaceae | -0.074335924 | 1 |
| IL10 | Day 6 | Peptococcaceae |  | 1 |
| IL10 | Day 6 | Peptostreptococcaceae | -0.060159211 | 1 |
| IL10 | Day 6 | Planococcaceae | -0.039755979 | 1 |
| IL10 | Day 6 | Porphyromonadaceae | 0.080093186 | 1 |
| IL10 | Day 6 | Prevotellaceae |  | 1 |
| IL10 | Day 6 | Pseudomonadaceae | 0.117791292 | 1 |
| IL10 | Day 6 | RFP12 |  | 1 |
| IL10 | Day 6 | Rikenellaceae | -0.132515204 | 1 |
| IL10 | Day 6 | Ruminococcaceae | 0.130870032 | 1 |
| IL10 | Day 6 | Spirochaetaceae |  | 1 |
| IL10 | Day 6 | Staphylococcaceae | -0.071024313 | 1 |
| IL10 | Day 6 | Streptococcaceae | -0.033976496 | 1 |
| IL10 | Day 6 | Succinivibrionaceae |  | 1 |
| IL10 | Day 6 | Tissierellaceae |  | 1 |
| IL10 | Day 6 | Turicibacteraceae |  | 1 |
| IL10 | Day 6 | Veillonellaceae | -0.263903469 | 1 |
| IL10 | Day 6 | Verrucomicrobiaceae |  | 1 |
| IL10 | Day 6 | Xanthomonadaceae |  | 1 |
| IL10 | Day 40 | Actinomycetaceae | -0.009265035 | 1 |
| IL10 | Day 40 | Aerococcaceae |  | 1 |
| IL10 | Day 40 | Aeromonadaceae |  | 1 |
| IL10 | Day 40 | Alcaligenaceae | -0.042859854 | 1 |
| IL10 | Day 40 | Bacillaceae |  | 1 |
| IL10 | Day 40 | Bacteroidaceae | -0.189123646 | 1 |
| IL10 | Day 40 | Barnesiellaceae | 0.124345877 | 1 |
| IL10 | Day 40 | Bifidobacteriaceae | -0.125269 | 1 |
| IL10 | Day 40 | BS11 |  | 1 |
| IL10 | Day 40 | Burkholderiaceae |  | 1 |
| IL10 | Day 40 | Campylobacteraceae |  | 1 |
| IL10 | Day 40 | Carnobacteriaceae |  | 1 |
| IL10 | Day 40 | Christensenellaceae |  | 1 |
| IL10 | Day 40 | Clostridiaceae | 0.051232644 | 1 |
| IL10 | Day 40 | Comamonadaceae | 0.14325328 | 1 |
| IL10 | Day 40 | Coriobacteriaceae | -0.0542 | 1 |
| IL10 | Day 40 | Corynebacteriaceae | -0.072425497 | 1 |
| IL10 | Day 40 | Dermabacteraceae |  | 1 |
| IL10 | Day 40 | Desulfovibrionaceae | -0.065779712 | 1 |
| IL10 | Day 40 | Dietziaceae |  | 1 |
| IL10 | Day 40 | Enterobacteriaceae | 0.196805499 | 1 |
| IL10 | Day 40 | Enterococcaceae | -0.249631655 | 1 |
| IL10 | Day 40 | Erysipelotrichaceae | -0.144815881 | 1 |
| IL10 | Day 40 | Eubacteriaceae | -0.174084228 | 1 |
| IL10 | Day 40 | Fibrobacteraceae |  | 1 |
| IL10 | Day 40 | Flavobacteriaceae |  | 1 |
| IL10 | Day 40 | Fusobacteriaceae |  | 1 |
| IL10 | Day 40 | Gemellaceae | 0.290344615 | 1 |
| IL10 | Day 40 | Helicobacteraceae |  | 1 |
| IL10 | Day 40 | Lachnospiraceae | -0.095245091 | 1 |
| IL10 | Day 40 | Lactobacillaceae | -0.120082811 | 1 |
| IL10 | Day 40 | Leptotrichiaceae |  | 1 |
| IL10 | Day 40 | Leuconostocaceae |  | 1 |
| IL10 | Day 40 | Micrococcaceae | 0.140987488 | 1 |
| IL10 | Day 40 | mitochondria |  | 1 |
| IL10 | Day 40 | Mogibacteriaceae |  | 1 |
| IL10 | Day 40 | Moraxellaceae |  | 1 |
| IL10 | Day 40 | Neisseriaceae | 0.007034552 | 1 |
| IL10 | Day 40 | Odoribacteraceae | 0.211387991 | 1 |
| IL10 | Day 40 | Other | -0.261126342 | 1 |
| IL10 | Day 40 | Other | -0.062952934 | 1 |
| IL10 | Day 40 | Other | 0.179393253 | 1 |
| IL10 | Day 40 | Other | 0.204217997 | 1 |
| IL10 | Day 40 | Other | 0.211387991 | 1 |
| IL10 | Day 40 | Paenibacillaceae |  | 1 |
| IL10 | Day 40 | Paraprevotellaceae |  | 1 |
| IL10 | Day 40 | Pasteurellaceae | 0.1555134 | 1 |
| IL10 | Day 40 | Peptococcaceae |  | 1 |
| IL10 | Day 40 | Peptostreptococcaceae | 0.216805694 | 1 |
| IL10 | Day 40 | Planococcaceae | -0.048000472 | 1 |
| IL10 | Day 40 | Porphyromonadaceae | -0.030169898 | 1 |
| IL10 | Day 40 | Prevotellaceae |  | 1 |
| IL10 | Day 40 | Pseudomonadaceae |  | 1 |
| IL10 | Day 40 | RFP12 |  | 1 |
| IL10 | Day 40 | Rikenellaceae |  | 1 |
| IL10 | Day 40 | Ruminococcaceae | 0.158155368 | 1 |
| IL10 | Day 40 | Spirochaetaceae |  | 1 |
| IL10 | Day 40 | Staphylococcaceae | 0.259697111 | 1 |
| IL10 | Day 40 | Streptococcaceae | 0.19058406 | 1 |
| IL10 | Day 40 | Succinivibrionaceae |  | 1 |
| IL10 | Day 40 | Tissierellaceae | -0.103409581 | 1 |
| IL10 | Day 40 | Turicibacteraceae |  | 1 |
| IL10 | Day 40 | Veillonellaceae | 0.265749128 | 1 |
| IL10 | Day 40 | Verrucomicrobiaceae | 0.211387991 | 1 |
| IL10 | Day 40 | Xanthomonadaceae |  | 1 |
| IL10 | Day 60C | Actinomycetaceae | -0.213783096 | 0.760857892 |
| IL10 | Day 60C | Aerococcaceae |  | 1 |
| IL10 | Day 60C | Aeromonadaceae |  | 1 |
| IL10 | Day 60C | Alcaligenaceae | 0.060592385 | 1 |
| IL10 | Day 60C | Bacillaceae |  | 1 |
| IL10 | Day 60C | Bacteroidaceae | 0.0433303 | 1 |
| IL10 | Day 60C | Barnesiellaceae |  | 1 |
| IL10 | Day 60C | Bifidobacteriaceae | -0.294322907 | 0.760857892 |
| IL10 | Day 60C | BS11 |  | 1 |
| IL10 | Day 60C | Burkholderiaceae |  | 1 |
| IL10 | Day 60C | Campylobacteraceae |  | 1 |
| IL10 | Day 60C | Carnobacteriaceae | -0.238578726 | 0.760857892 |
| IL10 | Day 60C | Christensenellaceae | -0.238578726 | 0.760857892 |
| IL10 | Day 60C | Clostridiaceae | 0.29763077 | 0.760857892 |
| IL10 | Day 60C | Comamonadaceae | -0.044828952 | 1 |
| IL10 | Day 60C | Coriobacteriaceae | -0.05950378 | 1 |
| IL10 | Day 60C | Corynebacteriaceae | 0.031985601 | 1 |
| IL10 | Day 60C | Dermabacteraceae |  | 1 |
| IL10 | Day 60C | Desulfovibrionaceae | -0.117337281 | 1 |
| IL10 | Day 60C | Dietziaceae |  | 1 |
| IL10 | Day 60C | Enterobacteriaceae | 0.278782994 | 0.760857892 |
| IL10 | Day 60C | Enterococcaceae | -0.15025043 | 1 |
| IL10 | Day 60C | Erysipelotrichaceae | 0.140397322 | 1 |
| IL10 | Day 60C | Eubacteriaceae | -0.186994137 | 0.911854061 |
| IL10 | Day 60C | Fibrobacteraceae |  | 1 |
| IL10 | Day 60C | Flavobacteriaceae |  | 1 |
| IL10 | Day 60C | Fusobacteriaceae | -0.096721105 | 1 |
| IL10 | Day 60C | Gemellaceae | 0.228347394 | 0.760857892 |
| IL10 | Day 60C | Helicobacteraceae |  | 1 |
| IL10 | Day 60C | Lachnospiraceae | -0.216079139 | 0.760857892 |
| IL10 | Day 60C | Lactobacillaceae | 0.104944556 | 1 |
| IL10 | Day 60C | Leptotrichiaceae |  | 1 |
| IL10 | Day 60C | Leuconostocaceae |  | 1 |
| IL10 | Day 60C | Micrococcaceae | 0.01326458 | 1 |
| IL10 | Day 60C | mitochondria |  | 1 |
| IL10 | Day 60C | Mogibacteriaceae | -0.238578726 | 0.760857892 |
| IL10 | Day 60C | Moraxellaceae |  | 1 |
| IL10 | Day 60C | Neisseriaceae | 0.212786431 | 0.760857892 |
| IL10 | Day 60C | Odoribacteraceae | -0.238578726 | 0.760857892 |
| IL10 | Day 60C | Other | -0.315223674 | 0.760857892 |
| IL10 | Day 60C | Other | 0.105317873 | 1 |
| IL10 | Day 60C | Other | 0.155357143 | 1 |
| IL10 | Day 60C | Other |  | 1 |
| IL10 | Day 60C | Other |  | 1 |
| IL10 | Day 60C | Paenibacillaceae |  | 1 |
| IL10 | Day 60C | Paraprevotellaceae |  | 1 |
| IL10 | Day 60C | Pasteurellaceae | -0.092733042 | 1 |
| IL10 | Day 60C | Peptococcaceae |  | 1 |
| IL10 | Day 60C | Peptostreptococcaceae | 0.149668175 | 1 |
| IL10 | Day 60C | Planococcaceae | -0.161820735 | 1 |
| IL10 | Day 60C | Porphyromonadaceae | -0.004859086 | 1 |
| IL10 | Day 60C | Prevotellaceae | -0.215942597 | 0.760857892 |
| IL10 | Day 60C | Pseudomonadaceae |  | 1 |
| IL10 | Day 60C | RFP12 |  | 1 |
| IL10 | Day 60C | Rikenellaceae |  | 1 |
| IL10 | Day 60C | Ruminococcaceae | -0.194250454 | 0.888903915 |
| IL10 | Day 60C | Spirochaetaceae |  | 1 |
| IL10 | Day 60C | Staphylococcaceae | 0.219639293 | 0.760857892 |
| IL10 | Day 60C | Streptococcaceae | 0.347120982 | 0.760857892 |
| IL10 | Day 60C | Succinivibrionaceae |  | 1 |
| IL10 | Day 60C | Tissierellaceae | -0.136273693 | 1 |
| IL10 | Day 60C | Turicibacteraceae |  | 1 |
| IL10 | Day 60C | Veillonellaceae | 0.172778925 | 0.958527012 |
| IL10 | Day 60C | Verrucomicrobiaceae | -0.019344221 | 1 |
| IL10 | Day 60C | Xanthomonadaceae |  | 1 |
| IL1B | Day 6 | Actinomycetaceae | 0.10033155 | 1 |
| IL1B | Day 6 | Aerococcaceae |  | 1 |
| IL1B | Day 6 | Aeromonadaceae |  | 1 |
| IL1B | Day 6 | Alcaligenaceae | -0.055485637 | 1 |
| IL1B | Day 6 | Bacillaceae |  | 1 |
| IL1B | Day 6 | Bacteroidaceae | 0.038064773 | 1 |
| IL1B | Day 6 | Barnesiellaceae |  | 1 |
| IL1B | Day 6 | Bifidobacteriaceae | -0.087804878 | 1 |
| IL1B | Day 6 | BS11 |  | 1 |
| IL1B | Day 6 | Burkholderiaceae |  | 1 |
| IL1B | Day 6 | Campylobacteraceae |  | 1 |
| IL1B | Day 6 | Carnobacteriaceae |  | 1 |
| IL1B | Day 6 | Christensenellaceae |  | 1 |
| IL1B | Day 6 | Clostridiaceae | -0.057291116 | 1 |
| IL1B | Day 6 | Comamonadaceae | -0.049932446 | 1 |
| IL1B | Day 6 | Coriobacteriaceae | -0.006253021 | 1 |
| IL1B | Day 6 | Corynebacteriaceae | 0.08897005 | 1 |
| IL1B | Day 6 | Dermabacteraceae |  | 1 |
| IL1B | Day 6 | Desulfovibrionaceae | -0.129204913 | 1 |
| IL1B | Day 6 | Dietziaceae |  | 1 |
| IL1B | Day 6 | Enterobacteriaceae | -0.177680974 | 1 |
| IL1B | Day 6 | Enterococcaceae | -0.096843864 | 1 |
| IL1B | Day 6 | Erysipelotrichaceae | -0.048758635 | 1 |
| IL1B | Day 6 | Eubacteriaceae | 0.06681531 | 1 |
| IL1B | Day 6 | Fibrobacteraceae |  | 1 |
| IL1B | Day 6 | Flavobacteriaceae |  | 1 |
| IL1B | Day 6 | Fusobacteriaceae |  | 1 |
| IL1B | Day 6 | Gemellaceae | 0.205361667 | 1 |
| IL1B | Day 6 | Helicobacteraceae |  | 1 |
| IL1B | Day 6 | Lachnospiraceae | -0.092676806 | 1 |
| IL1B | Day 6 | Lactobacillaceae | -0.222708469 | 1 |
| IL1B | Day 6 | Leptotrichiaceae |  | 1 |
| IL1B | Day 6 | Leuconostocaceae |  | 1 |
| IL1B | Day 6 | Micrococcaceae | 0.029457928 | 1 |
| IL1B | Day 6 | mitochondria |  | 1 |
| IL1B | Day 6 | Mogibacteriaceae |  | 1 |
| IL1B | Day 6 | Moraxellaceae |  | 1 |
| IL1B | Day 6 | Neisseriaceae | 0.093790941 | 1 |
| IL1B | Day 6 | Odoribacteraceae | -0.267261242 | 1 |
| IL1B | Day 6 | Other | -0.144285048 | 1 |
| IL1B | Day 6 | Other | -0.036139457 | 1 |
| IL1B | Day 6 | Other | 0.245982143 | 1 |
| IL1B | Day 6 | Other |  | 1 |
| IL1B | Day 6 | Other |  | 1 |
| IL1B | Day 6 | Paenibacillaceae |  | 1 |
| IL1B | Day 6 | Paraprevotellaceae |  | 1 |
| IL1B | Day 6 | Pasteurellaceae | 0.30781675 | 1 |
| IL1B | Day 6 | Peptococcaceae |  | 1 |
| IL1B | Day 6 | Peptostreptococcaceae | -0.171263622 | 1 |
| IL1B | Day 6 | Planococcaceae | 0.018990292 | 1 |
| IL1B | Day 6 | Porphyromonadaceae | -0.05137929 | 1 |
| IL1B | Day 6 | Prevotellaceae |  | 1 |
| IL1B | Day 6 | Pseudomonadaceae | -0.160356745 | 1 |
| IL1B | Day 6 | RFP12 |  | 1 |
| IL1B | Day 6 | Rikenellaceae | -0.267261242 | 1 |
| IL1B | Day 6 | Ruminococcaceae | 0.033480044 | 1 |
| IL1B | Day 6 | Spirochaetaceae |  | 1 |
| IL1B | Day 6 | Staphylococcaceae | 0.22195122 | 1 |
| IL1B | Day 6 | Streptococcaceae | 0.18554007 | 1 |
| IL1B | Day 6 | Succinivibrionaceae |  | 1 |
| IL1B | Day 6 | Tissierellaceae |  | 1 |
| IL1B | Day 6 | Turicibacteraceae |  | 1 |
| IL1B | Day 6 | Veillonellaceae | 0.106601652 | 1 |
| IL1B | Day 6 | Verrucomicrobiaceae |  | 1 |
| IL1B | Day 6 | Xanthomonadaceae |  | 1 |
| IL1B | Day 40 | Actinomycetaceae | 0.088267182 | 1 |
| IL1B | Day 40 | Aerococcaceae |  | 1 |
| IL1B | Day 40 | Aeromonadaceae |  | 1 |
| IL1B | Day 40 | Alcaligenaceae | 0.015789876 | 1 |
| IL1B | Day 40 | Bacillaceae |  | 1 |
| IL1B | Day 40 | Bacteroidaceae | 0.184981975 | 1 |
| IL1B | Day 40 | Barnesiellaceae | 0.186511774 | 1 |
| IL1B | Day 40 | Bifidobacteriaceae | -0.739806705 | 1.37E-07 |
| IL1B | Day 40 | BS11 |  | 1 |
| IL1B | Day 40 | Burkholderiaceae |  | 1 |
| IL1B | Day 40 | Campylobacteraceae |  | 1 |
| IL1B | Day 40 | Carnobacteriaceae |  | 1 |
| IL1B | Day 40 | Christensenellaceae |  | 1 |
| IL1B | Day 40 | Clostridiaceae | 0.561840409 | 0.001064952 |
| IL1B | Day 40 | Comamonadaceae | 0.119658127 | 1 |
| IL1B | Day 40 | Coriobacteriaceae | 0.178334109 | 1 |
| IL1B | Day 40 | Corynebacteriaceae | -0.18458311 | 1 |
| IL1B | Day 40 | Dermabacteraceae |  | 1 |
| IL1B | Day 40 | Desulfovibrionaceae | 0.170207056 | 1 |
| IL1B | Day 40 | Dietziaceae |  | 1 |
| IL1B | Day 40 | Enterobacteriaceae | 0.714393604 | 5.29E-07 |
| IL1B | Day 40 | Enterococcaceae | 0.113347712 | 1 |
| IL1B | Day 40 | Erysipelotrichaceae | 0.231441864 | 1 |
| IL1B | Day 40 | Eubacteriaceae | -0.136775301 | 1 |
| IL1B | Day 40 | Fibrobacteraceae |  | 1 |
| IL1B | Day 40 | Flavobacteriaceae |  | 1 |
| IL1B | Day 40 | Fusobacteriaceae |  | 1 |
| IL1B | Day 40 | Gemellaceae | 0.131752727 | 1 |
| IL1B | Day 40 | Helicobacteraceae |  | 1 |
| IL1B | Day 40 | Lachnospiraceae | 0.173912119 | 1 |
| IL1B | Day 40 | Lactobacillaceae | -0.050922828 | 1 |
| IL1B | Day 40 | Leptotrichiaceae |  | 1 |
| IL1B | Day 40 | Leuconostocaceae |  | 1 |
| IL1B | Day 40 | Micrococcaceae | 0.050706191 | 1 |
| IL1B | Day 40 | mitochondria |  | 1 |
| IL1B | Day 40 | Mogibacteriaceae |  | 1 |
| IL1B | Day 40 | Moraxellaceae |  | 1 |
| IL1B | Day 40 | Neisseriaceae | -0.237096827 | 1 |
| IL1B | Day 40 | Odoribacteraceae | -0.07460471 | 1 |
| IL1B | Day 40 | Other | 0.607677369 | 0.000199184 |
| IL1B | Day 40 | Other | -0.087038828 | 1 |
| IL1B | Day 40 | Other | -0.07460471 | 1 |
| IL1B | Day 40 | Other | 0.048714389 | 1 |
| IL1B | Day 40 | Other | 0.067627737 | 1 |
| IL1B | Day 40 | Paenibacillaceae |  | 1 |
| IL1B | Day 40 | Paraprevotellaceae |  | 1 |
| IL1B | Day 40 | Pasteurellaceae | 0.182776262 | 1 |
| IL1B | Day 40 | Peptococcaceae |  | 1 |
| IL1B | Day 40 | Peptostreptococcaceae | 0.403519063 | 0.099445097 |
| IL1B | Day 40 | Planococcaceae | 0.058343198 | 1 |
| IL1B | Day 40 | Porphyromonadaceae | 0.051554209 | 1 |
| IL1B | Day 40 | Prevotellaceae |  | 1 |
| IL1B | Day 40 | Pseudomonadaceae |  | 1 |
| IL1B | Day 40 | RFP12 |  | 1 |
| IL1B | Day 40 | Rikenellaceae |  | 1 |
| IL1B | Day 40 | Ruminococcaceae | 0.199503835 | 1 |
| IL1B | Day 40 | Spirochaetaceae |  | 1 |
| IL1B | Day 40 | Staphylococcaceae | 0.358410782 | 0.215198067 |
| IL1B | Day 40 | Streptococcaceae | 0.209302326 | 1 |
| IL1B | Day 40 | Succinivibrionaceae |  | 1 |
| IL1B | Day 40 | Tissierellaceae | -0.053826158 | 1 |
| IL1B | Day 40 | Turicibacteraceae |  | 1 |
| IL1B | Day 40 | Veillonellaceae | 0.256053843 | 1 |
| IL1B | Day 40 | Verrucomicrobiaceae | -0.07460471 | 1 |
| IL1B | Day 40 | Xanthomonadaceae |  | 1 |
| IL1B | Day 60C | Actinomycetaceae | -0.127583816 | 1 |
| IL1B | Day 60C | Aerococcaceae |  | 1 |
| IL1B | Day 60C | Aeromonadaceae |  | 1 |
| IL1B | Day 60C | Alcaligenaceae | 0.150872925 | 1 |
| IL1B | Day 60C | Bacillaceae |  | 1 |
| IL1B | Day 60C | Bacteroidaceae | 0.355704854 | 0.245585561 |
| IL1B | Day 60C | Barnesiellaceae |  | 1 |
| IL1B | Day 60C | Bifidobacteriaceae | -0.636010048 | 9.84E-05 |
| IL1B | Day 60C | BS11 |  | 1 |
| IL1B | Day 60C | Burkholderiaceae |  | 1 |
| IL1B | Day 60C | Campylobacteraceae |  | 1 |
| IL1B | Day 60C | Carnobacteriaceae | 0.096634856 | 1 |
| IL1B | Day 60C | Christensenellaceae | 0.096634856 | 1 |
| IL1B | Day 60C | Clostridiaceae | 0.63766561 | 9.84E-05 |
| IL1B | Day 60C | Comamonadaceae | -0.120315485 | 1 |
| IL1B | Day 60C | Coriobacteriaceae | -0.043252805 | 1 |
| IL1B | Day 60C | Corynebacteriaceae | -0.053960312 | 1 |
| IL1B | Day 60C | Dermabacteraceae |  | 1 |
| IL1B | Day 60C | Desulfovibrionaceae | -0.073761371 | 1 |
| IL1B | Day 60C | Dietziaceae |  | 1 |
| IL1B | Day 60C | Enterobacteriaceae | 0.57214661 | 0.001175462 |
| IL1B | Day 60C | Enterococcaceae | 0.007930989 | 1 |
| IL1B | Day 60C | Erysipelotrichaceae | 0.109353675 | 1 |
| IL1B | Day 60C | Eubacteriaceae | 0.045096266 | 1 |
| IL1B | Day 60C | Fibrobacteraceae |  | 1 |
| IL1B | Day 60C | Flavobacteriaceae |  | 1 |
| IL1B | Day 60C | Fusobacteriaceae | 0.264135272 | 0.950138336 |
| IL1B | Day 60C | Gemellaceae | -0.247285672 | 1 |
| IL1B | Day 60C | Helicobacteraceae |  | 1 |
| IL1B | Day 60C | Lachnospiraceae | -0.107145617 | 1 |
| IL1B | Day 60C | Lactobacillaceae | -0.079134594 | 1 |
| IL1B | Day 60C | Leptotrichiaceae |  | 1 |
| IL1B | Day 60C | Leuconostocaceae |  | 1 |
| IL1B | Day 60C | Micrococcaceae | -0.126871881 | 1 |
| IL1B | Day 60C | mitochondria |  | 1 |
| IL1B | Day 60C | Mogibacteriaceae | 0.096634856 | 1 |
| IL1B | Day 60C | Moraxellaceae |  | 1 |
| IL1B | Day 60C | Neisseriaceae | -0.057980913 | 1 |
| IL1B | Day 60C | Odoribacteraceae | 0.096634856 | 1 |
| IL1B | Day 60C | Other | 0.372638189 | 0.245585561 |
| IL1B | Day 60C | Other | -0.214290826 | 1 |
| IL1B | Day 60C | Other | 0.098788586 | 1 |
| IL1B | Day 60C | Other |  | 1 |
| IL1B | Day 60C | Other |  | 1 |
| IL1B | Day 60C | Paenibacillaceae |  | 1 |
| IL1B | Day 60C | Paraprevotellaceae |  | 1 |
| IL1B | Day 60C | Pasteurellaceae | 0.014472806 | 1 |
| IL1B | Day 60C | Peptococcaceae |  | 1 |
| IL1B | Day 60C | Peptostreptococcaceae | 0.033406691 | 1 |
| IL1B | Day 60C | Planococcaceae | 0.006350926 | 1 |
| IL1B | Day 60C | Porphyromonadaceae | -0.096982167 | 1 |
| IL1B | Day 60C | Prevotellaceae | 0.190709239 | 1 |
| IL1B | Day 60C | Pseudomonadaceae |  | 1 |
| IL1B | Day 60C | RFP12 |  | 1 |
| IL1B | Day 60C | Rikenellaceae |  | 1 |
| IL1B | Day 60C | Ruminococcaceae | 0.053176485 | 1 |
| IL1B | Day 60C | Spirochaetaceae |  | 1 |
| IL1B | Day 60C | Staphylococcaceae | 0.361976084 | 0.245585561 |
| IL1B | Day 60C | Streptococcaceae | 0.06960538 | 1 |
| IL1B | Day 60C | Succinivibrionaceae |  | 1 |
| IL1B | Day 60C | Tissierellaceae | 0.145079215 | 1 |
| IL1B | Day 60C | Turicibacteraceae |  | 1 |
| IL1B | Day 60C | Veillonellaceae | 0.152399494 | 1 |
| IL1B | Day 60C | Verrucomicrobiaceae | -0.045096266 | 1 |
| IL1B | Day 60C | Xanthomonadaceae |  | 1 |
| IL2 | Day 6 | Actinomycetaceae | -0.221761564 | 1 |
| IL2 | Day 6 | Aerococcaceae |  | 1 |
| IL2 | Day 6 | Aeromonadaceae |  | 1 |
| IL2 | Day 6 | Alcaligenaceae | 0.245265148 | 1 |
| IL2 | Day 6 | Bacillaceae |  | 1 |
| IL2 | Day 6 | Bacteroidaceae | 0.123123058 | 1 |
| IL2 | Day 6 | Barnesiellaceae |  | 1 |
| IL2 | Day 6 | Bifidobacteriaceae | 0.024047508 | 1 |
| IL2 | Day 6 | BS11 |  | 1 |
| IL2 | Day 6 | Burkholderiaceae |  | 1 |
| IL2 | Day 6 | Campylobacteraceae |  | 1 |
| IL2 | Day 6 | Carnobacteriaceae |  | 1 |
| IL2 | Day 6 | Christensenellaceae |  | 1 |
| IL2 | Day 6 | Clostridiaceae | -0.08719645 | 1 |
| IL2 | Day 6 | Comamonadaceae | -0.118502299 | 1 |
| IL2 | Day 6 | Coriobacteriaceae | -0.199688687 | 1 |
| IL2 | Day 6 | Corynebacteriaceae | 0.054337074 | 1 |
| IL2 | Day 6 | Dermabacteraceae |  | 1 |
| IL2 | Day 6 | Desulfovibrionaceae | -0.044484748 | 1 |
| IL2 | Day 6 | Dietziaceae |  | 1 |
| IL2 | Day 6 | Enterobacteriaceae | 0.261997377 | 1 |
| IL2 | Day 6 | Enterococcaceae | -0.155582773 | 1 |
| IL2 | Day 6 | Erysipelotrichaceae | 0.107232127 | 1 |
| IL2 | Day 6 | Eubacteriaceae | 0.154884995 | 1 |
| IL2 | Day 6 | Fibrobacteraceae |  | 1 |
| IL2 | Day 6 | Flavobacteriaceae |  | 1 |
| IL2 | Day 6 | Fusobacteriaceae |  | 1 |
| IL2 | Day 6 | Gemellaceae | 0.061439623 | 1 |
| IL2 | Day 6 | Helicobacteraceae |  | 1 |
| IL2 | Day 6 | Lachnospiraceae | -0.189977786 | 1 |
| IL2 | Day 6 | Lactobacillaceae | 0.043589081 | 1 |
| IL2 | Day 6 | Leptotrichiaceae |  | 1 |
| IL2 | Day 6 | Leuconostocaceae |  | 1 |
| IL2 | Day 6 | Micrococcaceae | -0.131742655 | 1 |
| IL2 | Day 6 | mitochondria |  | 1 |
| IL2 | Day 6 | Mogibacteriaceae |  | 1 |
| IL2 | Day 6 | Moraxellaceae |  | 1 |
| IL2 | Day 6 | Neisseriaceae | 0.151185005 | 1 |
| IL2 | Day 6 | Odoribacteraceae | 0 | 1 |
| IL2 | Day 6 | Other | -0.269431084 | 1 |
| IL2 | Day 6 | Other | -0.192069632 | 1 |
| IL2 | Day 6 | Other | 0.028626692 | 1 |
| IL2 | Day 6 | Other |  | 1 |
| IL2 | Day 6 | Other |  | 1 |
| IL2 | Day 6 | Paenibacillaceae |  | 1 |
| IL2 | Day 6 | Paraprevotellaceae |  | 1 |
| IL2 | Day 6 | Pasteurellaceae | -0.003933686 | 1 |
| IL2 | Day 6 | Peptococcaceae |  | 1 |
| IL2 | Day 6 | Peptostreptococcaceae | -0.118502299 | 1 |
| IL2 | Day 6 | Planococcaceae | 0.033794295 | 1 |
| IL2 | Day 6 | Porphyromonadaceae | -0.058473024 | 1 |
| IL2 | Day 6 | Prevotellaceae |  | 1 |
| IL2 | Day 6 | Pseudomonadaceae | 0.112643633 | 1 |
| IL2 | Day 6 | RFP12 |  | 1 |
| IL2 | Day 6 | Rikenellaceae | 0 | 1 |
| IL2 | Day 6 | Ruminococcaceae | 0.001539887 | 1 |
| IL2 | Day 6 | Spirochaetaceae |  | 1 |
| IL2 | Day 6 | Staphylococcaceae | -0.073794643 | 1 |
| IL2 | Day 6 | Streptococcaceae | -0.087195163 | 1 |
| IL2 | Day 6 | Succinivibrionaceae |  | 1 |
| IL2 | Day 6 | Tissierellaceae |  | 1 |
| IL2 | Day 6 | Turicibacteraceae |  | 1 |
| IL2 | Day 6 | Veillonellaceae | -0.221579558 | 1 |
| IL2 | Day 6 | Verrucomicrobiaceae |  | 1 |
| IL2 | Day 6 | Xanthomonadaceae |  | 1 |
| IL2 | Day 40 | Actinomycetaceae | -0.076981111 | 1 |
| IL2 | Day 40 | Aerococcaceae |  | 1 |
| IL2 | Day 40 | Aeromonadaceae |  | 1 |
| IL2 | Day 40 | Alcaligenaceae | -0.054637985 | 1 |
| IL2 | Day 40 | Bacillaceae |  | 1 |
| IL2 | Day 40 | Bacteroidaceae | -0.0099535 | 1 |
| IL2 | Day 40 | Barnesiellaceae | -0.012434118 | 1 |
| IL2 | Day 40 | Bifidobacteriaceae | 0.043189369 | 1 |
| IL2 | Day 40 | BS11 |  | 1 |
| IL2 | Day 40 | Burkholderiaceae |  | 1 |
| IL2 | Day 40 | Campylobacteraceae |  | 1 |
| IL2 | Day 40 | Carnobacteriaceae |  | 1 |
| IL2 | Day 40 | Christensenellaceae |  | 1 |
| IL2 | Day 40 | Clostridiaceae | 0.044441098 | 1 |
| IL2 | Day 40 | Comamonadaceae | 0.164273514 | 1 |
| IL2 | Day 40 | Coriobacteriaceae | 0.028822513 | 1 |
| IL2 | Day 40 | Corynebacteriaceae | 0.381372303 | 0.808414532 |
| IL2 | Day 40 | Dermabacteraceae |  | 1 |
| IL2 | Day 40 | Desulfovibrionaceae | -0.069303431 | 1 |
| IL2 | Day 40 | Dietziaceae |  | 1 |
| IL2 | Day 40 | Enterobacteriaceae | 0.014046217 | 1 |
| IL2 | Day 40 | Enterococcaceae | -0.243644615 | 1 |
| IL2 | Day 40 | Erysipelotrichaceae | -0.092045916 | 1 |
| IL2 | Day 40 | Eubacteriaceae | -0.174077656 | 1 |
| IL2 | Day 40 | Fibrobacteraceae |  | 1 |
| IL2 | Day 40 | Flavobacteriaceae |  | 1 |
| IL2 | Day 40 | Fusobacteriaceae |  | 1 |
| IL2 | Day 40 | Gemellaceae | 0.168930083 | 1 |
| IL2 | Day 40 | Helicobacteraceae |  | 1 |
| IL2 | Day 40 | Lachnospiraceae | -0.08104961 | 1 |
| IL2 | Day 40 | Lactobacillaceae | -0.075661363 | 1 |
| IL2 | Day 40 | Leptotrichiaceae |  | 1 |
| IL2 | Day 40 | Leuconostocaceae |  | 1 |
| IL2 | Day 40 | Micrococcaceae | 0.038700987 | 1 |
| IL2 | Day 40 | mitochondria |  | 1 |
| IL2 | Day 40 | Mogibacteriaceae |  | 1 |
| IL2 | Day 40 | Moraxellaceae |  | 1 |
| IL2 | Day 40 | Neisseriaceae | -0.036826558 | 1 |
| IL2 | Day 40 | Odoribacteraceae | 0.198945893 | 1 |
| IL2 | Day 40 | Other | -0.016986559 | 1 |
| IL2 | Day 40 | Other | 0.024868237 | 1 |
| IL2 | Day 40 | Other | 0.043373142 | 1 |
| IL2 | Day 40 | Other | 0.198945893 | 1 |
| IL2 | Day 40 | Other | 0.293176096 | 1 |
| IL2 | Day 40 | Paenibacillaceae |  | 1 |
| IL2 | Day 40 | Paraprevotellaceae |  | 1 |
| IL2 | Day 40 | Pasteurellaceae | -0.088320398 | 1 |
| IL2 | Day 40 | Peptococcaceae |  | 1 |
| IL2 | Day 40 | Peptostreptococcaceae | 0.304769677 | 1 |
| IL2 | Day 40 | Planococcaceae | 0.043033281 | 1 |
| IL2 | Day 40 | Porphyromonadaceae | -0.066638589 | 1 |
| IL2 | Day 40 | Prevotellaceae |  | 1 |
| IL2 | Day 40 | Pseudomonadaceae |  | 1 |
| IL2 | Day 40 | RFP12 |  | 1 |
| IL2 | Day 40 | Rikenellaceae |  | 1 |
| IL2 | Day 40 | Ruminococcaceae | 0.037845577 | 1 |
| IL2 | Day 40 | Spirochaetaceae |  | 1 |
| IL2 | Day 40 | Staphylococcaceae | -0.040939651 | 1 |
| IL2 | Day 40 | Streptococcaceae | 0.241769858 | 1 |
| IL2 | Day 40 | Succinivibrionaceae |  | 1 |
| IL2 | Day 40 | Tissierellaceae | -0.193115941 | 1 |
| IL2 | Day 40 | Turicibacteraceae |  | 1 |
| IL2 | Day 40 | Veillonellaceae | -0.039951664 | 1 |
| IL2 | Day 40 | Verrucomicrobiaceae | 0.198945893 | 1 |
| IL2 | Day 40 | Xanthomonadaceae |  | 1 |
| IL2 | Day 60C | Actinomycetaceae | -0.268961203 | 0.776228381 |
| IL2 | Day 60C | Aerococcaceae |  | 1 |
| IL2 | Day 60C | Aeromonadaceae |  | 1 |
| IL2 | Day 60C | Alcaligenaceae | -0.128477051 | 1 |
| IL2 | Day 60C | Bacillaceae |  | 1 |
| IL2 | Day 60C | Bacteroidaceae | -0.127894926 | 1 |
| IL2 | Day 60C | Barnesiellaceae |  | 1 |
| IL2 | Day 60C | Bifidobacteriaceae | -0.329659645 | 0.592576754 |
| IL2 | Day 60C | BS11 |  | 1 |
| IL2 | Day 60C | Burkholderiaceae |  | 1 |
| IL2 | Day 60C | Campylobacteraceae |  | 1 |
| IL2 | Day 60C | Carnobacteriaceae | -0.083756995 | 1 |
| IL2 | Day 60C | Christensenellaceae | -0.083756995 | 1 |
| IL2 | Day 60C | Clostridiaceae | 0.314812528 | 0.608750749 |
| IL2 | Day 60C | Comamonadaceae | -0.052477612 | 1 |
| IL2 | Day 60C | Coriobacteriaceae | -0.303888715 | 0.608750749 |
| IL2 | Day 60C | Corynebacteriaceae | 0.066748552 | 1 |
| IL2 | Day 60C | Dermabacteraceae |  | 1 |
| IL2 | Day 60C | Desulfovibrionaceae | -0.283152105 | 0.720313572 |
| IL2 | Day 60C | Dietziaceae |  | 1 |
| IL2 | Day 60C | Enterobacteriaceae | 0.368625881 | 0.573130278 |
| IL2 | Day 60C | Enterococcaceae | 0.074164843 | 1 |
| IL2 | Day 60C | Erysipelotrichaceae | 0.072203143 | 1 |
| IL2 | Day 60C | Eubacteriaceae | -0.019328537 | 1 |
| IL2 | Day 60C | Fibrobacteraceae |  | 1 |
| IL2 | Day 60C | Flavobacteriaceae |  | 1 |
| IL2 | Day 60C | Fusobacteriaceae | -0.096642687 | 1 |
| IL2 | Day 60C | Gemellaceae | 0.050413984 | 1 |
| IL2 | Day 60C | Helicobacteraceae |  | 1 |
| IL2 | Day 60C | Lachnospiraceae | -0.334314326 | 0.592576754 |
| IL2 | Day 60C | Lactobacillaceae | -0.138331294 | 1 |
| IL2 | Day 60C | Leptotrichiaceae |  | 1 |
| IL2 | Day 60C | Leuconostocaceae |  | 1 |
| IL2 | Day 60C | Micrococcaceae | -0.119706525 | 1 |
| IL2 | Day 60C | mitochondria |  | 1 |
| IL2 | Day 60C | Mogibacteriaceae | -0.083756995 | 1 |
| IL2 | Day 60C | Moraxellaceae |  | 1 |
| IL2 | Day 60C | Neisseriaceae | 0.006442846 | 1 |
| IL2 | Day 60C | Odoribacteraceae | -0.083756995 | 1 |
| IL2 | Day 60C | Other | -0.101448488 | 1 |
| IL2 | Day 60C | Other | -0.033760207 | 1 |
| IL2 | Day 60C | Other | 0.162692663 | 1 |
| IL2 | Day 60C | Other |  | 1 |
| IL2 | Day 60C | Other |  | 1 |
| IL2 | Day 60C | Paenibacillaceae |  | 1 |
| IL2 | Day 60C | Paraprevotellaceae |  | 1 |
| IL2 | Day 60C | Pasteurellaceae | -0.052285215 | 1 |
| IL2 | Day 60C | Peptococcaceae |  | 1 |
| IL2 | Day 60C | Peptostreptococcaceae | 0.209206468 | 1 |
| IL2 | Day 60C | Planococcaceae | -0.113055646 | 1 |
| IL2 | Day 60C | Porphyromonadaceae | -0.383217868 | 0.573130278 |
| IL2 | Day 60C | Prevotellaceae | -0.21431576 | 1 |
| IL2 | Day 60C | Pseudomonadaceae |  | 1 |
| IL2 | Day 60C | RFP12 |  | 1 |
| IL2 | Day 60C | Rikenellaceae |  | 1 |
| IL2 | Day 60C | Ruminococcaceae | -0.220062352 | 1 |
| IL2 | Day 60C | Spirochaetaceae |  | 1 |
| IL2 | Day 60C | Staphylococcaceae | 0.218405333 | 1 |
| IL2 | Day 60C | Streptococcaceae | 0.176742302 | 1 |
| IL2 | Day 60C | Succinivibrionaceae |  | 1 |
| IL2 | Day 60C | Tissierellaceae | -0.025068693 | 1 |
| IL2 | Day 60C | Turicibacteraceae |  | 1 |
| IL2 | Day 60C | Veillonellaceae | -0.070693153 | 1 |
| IL2 | Day 60C | Verrucomicrobiaceae | -0.186842528 | 1 |
| IL2 | Day 60C | Xanthomonadaceae |  | 1 |
| IL22 | Day 6 | Actinomycetaceae | -0.093331674 | 1 |
| IL22 | Day 6 | Aerococcaceae |  | 1 |
| IL22 | Day 6 | Aeromonadaceae |  | 1 |
| IL22 | Day 6 | Alcaligenaceae | 0.310584236 | 1 |
| IL22 | Day 6 | Bacillaceae |  | 1 |
| IL22 | Day 6 | Bacteroidaceae | 0.103748637 | 1 |
| IL22 | Day 6 | Barnesiellaceae |  | 1 |
| IL22 | Day 6 | Bifidobacteriaceae | -0.140766551 | 1 |
| IL22 | Day 6 | BS11 |  | 1 |
| IL22 | Day 6 | Burkholderiaceae |  | 1 |
| IL22 | Day 6 | Campylobacteraceae |  | 1 |
| IL22 | Day 6 | Carnobacteriaceae |  | 1 |
| IL22 | Day 6 | Christensenellaceae |  | 1 |
| IL22 | Day 6 | Clostridiaceae | -0.111773845 | 1 |
| IL22 | Day 6 | Comamonadaceae | 0.034999378 | 1 |
| IL22 | Day 6 | Coriobacteriaceae | 0.113275888 | 1 |
| IL22 | Day 6 | Corynebacteriaceae | 0.023800905 | 1 |
| IL22 | Day 6 | Dermabacteraceae |  | 1 |
| IL22 | Day 6 | Desulfovibrionaceae | -0.180490197 | 1 |
| IL22 | Day 6 | Dietziaceae |  | 1 |
| IL22 | Day 6 | Enterobacteriaceae | 0.03888406 | 1 |
| IL22 | Day 6 | Enterococcaceae | -0.16128841 | 1 |
| IL22 | Day 6 | Erysipelotrichaceae | 0.186000244 | 1 |
| IL22 | Day 6 | Eubacteriaceae | -0.173719807 | 1 |
| IL22 | Day 6 | Fibrobacteraceae |  | 1 |
| IL22 | Day 6 | Flavobacteriaceae |  | 1 |
| IL22 | Day 6 | Fusobacteriaceae |  | 1 |
| IL22 | Day 6 | Gemellaceae | -0.097564435 | 1 |
| IL22 | Day 6 | Helicobacteraceae |  | 1 |
| IL22 | Day 6 | Lachnospiraceae | -0.034824012 | 1 |
| IL22 | Day 6 | Lactobacillaceae | 0.152722474 | 1 |
| IL22 | Day 6 | Leptotrichiaceae |  | 1 |
| IL22 | Day 6 | Leuconostocaceae |  | 1 |
| IL22 | Day 6 | Micrococcaceae | 0.07492354 | 1 |
| IL22 | Day 6 | mitochondria |  | 1 |
| IL22 | Day 6 | Mogibacteriaceae |  | 1 |
| IL22 | Day 6 | Moraxellaceae |  | 1 |
| IL22 | Day 6 | Neisseriaceae | 0.054966345 | 1 |
| IL22 | Day 6 | Odoribacteraceae | -0.25389818 | 1 |
| IL22 | Day 6 | Other | -0.307110541 | 1 |
| IL22 | Day 6 | Other | -0.241676532 | 1 |
| IL22 | Day 6 | Other | -0.169433969 | 1 |
| IL22 | Day 6 | Other |  | 1 |
| IL22 | Day 6 | Other |  | 1 |
| IL22 | Day 6 | Paenibacillaceae |  | 1 |
| IL22 | Day 6 | Paraprevotellaceae |  | 1 |
| IL22 | Day 6 | Pasteurellaceae | 0.069243213 | 1 |
| IL22 | Day 6 | Peptococcaceae |  | 1 |
| IL22 | Day 6 | Peptostreptococcaceae | 0.212329559 | 1 |
| IL22 | Day 6 | Planococcaceae | -0.174921688 | 1 |
| IL22 | Day 6 | Porphyromonadaceae | 0.00327055 | 1 |
| IL22 | Day 6 | Prevotellaceae |  | 1 |
| IL22 | Day 6 | Pseudomonadaceae | 0.06681531 | 1 |
| IL22 | Day 6 | RFP12 |  | 1 |
| IL22 | Day 6 | Rikenellaceae | -0.25389818 | 1 |
| IL22 | Day 6 | Ruminococcaceae | 0.148800195 | 1 |
| IL22 | Day 6 | Spirochaetaceae |  | 1 |
| IL22 | Day 6 | Staphylococcaceae | -0.1228223 | 1 |
| IL22 | Day 6 | Streptococcaceae | -0.186585366 | 1 |
| IL22 | Day 6 | Succinivibrionaceae |  | 1 |
| IL22 | Day 6 | Tissierellaceae |  | 1 |
| IL22 | Day 6 | Turicibacteraceae |  | 1 |
| IL22 | Day 6 | Veillonellaceae | -0.189807381 | 1 |
| IL22 | Day 6 | Verrucomicrobiaceae |  | 1 |
| IL22 | Day 6 | Xanthomonadaceae |  | 1 |
| IL22 | Day 40 | Actinomycetaceae | -0.076138867 | 1 |
| IL22 | Day 40 | Aerococcaceae |  | 1 |
| IL22 | Day 40 | Aeromonadaceae |  | 1 |
| IL22 | Day 40 | Alcaligenaceae | -0.026567094 | 1 |
| IL22 | Day 40 | Bacillaceae |  | 1 |
| IL22 | Day 40 | Bacteroidaceae | 0.074421556 | 1 |
| IL22 | Day 40 | Barnesiellaceae | 0.211380011 | 0.850064873 |
| IL22 | Day 40 | Bifidobacteriaceae | -0.483539716 | 0.020622467 |
| IL22 | Day 40 | BS11 |  | 1 |
| IL22 | Day 40 | Burkholderiaceae |  | 1 |
| IL22 | Day 40 | Campylobacteraceae |  | 1 |
| IL22 | Day 40 | Carnobacteriaceae |  | 1 |
| IL22 | Day 40 | Christensenellaceae |  | 1 |
| IL22 | Day 40 | Clostridiaceae | 0.432837778 | 0.061722529 |
| IL22 | Day 40 | Comamonadaceae | 0.23572651 | 0.670829091 |
| IL22 | Day 40 | Coriobacteriaceae | 0.020682437 | 1 |
| IL22 | Day 40 | Corynebacteriaceae | -0.379609202 | 0.167811856 |
| IL22 | Day 40 | Dermabacteraceae |  | 1 |
| IL22 | Day 40 | Desulfovibrionaceae | -0.086663194 | 1 |
| IL22 | Day 40 | Dietziaceae |  | 1 |
| IL22 | Day 40 | Enterobacteriaceae | 0.50853346 | 0.014249452 |
| IL22 | Day 40 | Enterococcaceae | 0.033141721 | 1 |
| IL22 | Day 40 | Erysipelotrichaceae | 0.187382977 | 0.999001865 |
| IL22 | Day 40 | Eubacteriaceae | -0.174077656 | 1 |
| IL22 | Day 40 | Fibrobacteraceae |  | 1 |
| IL22 | Day 40 | Flavobacteriaceae |  | 1 |
| IL22 | Day 40 | Fusobacteriaceae |  | 1 |
| IL22 | Day 40 | Gemellaceae | 0.136847697 | 1 |
| IL22 | Day 40 | Helicobacteraceae |  | 1 |
| IL22 | Day 40 | Lachnospiraceae | 0.139293763 | 1 |
| IL22 | Day 40 | Lactobacillaceae | -0.01012031 | 1 |
| IL22 | Day 40 | Leptotrichiaceae |  | 1 |
| IL22 | Day 40 | Leuconostocaceae |  | 1 |
| IL22 | Day 40 | Micrococcaceae | 0.089249214 | 1 |
| IL22 | Day 40 | mitochondria |  | 1 |
| IL22 | Day 40 | Mogibacteriaceae |  | 1 |
| IL22 | Day 40 | Moraxellaceae |  | 1 |
| IL22 | Day 40 | Neisseriaceae | -0.295440025 | 0.553063686 |
| IL22 | Day 40 | Odoribacteraceae | -0.261116484 | 0.553063686 |
| IL22 | Day 40 | Other | 0.320689226 | 0.432162136 |
| IL22 | Day 40 | Other | -0.261116484 | 0.553063686 |
| IL22 | Day 40 | Other | 0.186511774 | 0.999001865 |
| IL22 | Day 40 | Other | -0.003821976 | 1 |
| IL22 | Day 40 | Other | 0.086306772 | 1 |
| IL22 | Day 40 | Paenibacillaceae |  | 1 |
| IL22 | Day 40 | Paraprevotellaceae |  | 1 |
| IL22 | Day 40 | Pasteurellaceae | -0.017800423 | 1 |
| IL22 | Day 40 | Peptococcaceae |  | 1 |
| IL22 | Day 40 | Peptostreptococcaceae | 0.529336808 | 0.014169211 |
| IL22 | Day 40 | Planococcaceae | -0.010344539 | 1 |
| IL22 | Day 40 | Porphyromonadaceae | 0.049644794 | 1 |
| IL22 | Day 40 | Prevotellaceae |  | 1 |
| IL22 | Day 40 | Pseudomonadaceae |  | 1 |
| IL22 | Day 40 | RFP12 |  | 1 |
| IL22 | Day 40 | Rikenellaceae |  | 1 |
| IL22 | Day 40 | Ruminococcaceae | 0.085716472 | 1 |
| IL22 | Day 40 | Spirochaetaceae |  | 1 |
| IL22 | Day 40 | Staphylococcaceae | 0.179092089 | 1 |
| IL22 | Day 40 | Streptococcaceae | -0.02718212 | 1 |
| IL22 | Day 40 | Succinivibrionaceae |  | 1 |
| IL22 | Day 40 | Tissierellaceae | -0.129097847 | 1 |
| IL22 | Day 40 | Turicibacteraceae |  | 1 |
| IL22 | Day 40 | Veillonellaceae | 0.275424347 | 0.553063686 |
| IL22 | Day 40 | Verrucomicrobiaceae | -0.261116484 | 0.553063686 |
| IL22 | Day 40 | Xanthomonadaceae |  | 1 |
| IL22 | Day 60C | Actinomycetaceae | -0.227364234 | 1 |
| IL22 | Day 60C | Aerococcaceae |  | 1 |
| IL22 | Day 60C | Aeromonadaceae |  | 1 |
| IL22 | Day 60C | Alcaligenaceae | 0.116465385 | 1 |
| IL22 | Day 60C | Bacillaceae |  | 1 |
| IL22 | Day 60C | Bacteroidaceae | 0.134768102 | 1 |
| IL22 | Day 60C | Barnesiellaceae |  | 1 |
| IL22 | Day 60C | Bifidobacteriaceae | -0.511060692 | 0.015562168 |
| IL22 | Day 60C | BS11 |  | 1 |
| IL22 | Day 60C | Burkholderiaceae |  | 1 |
| IL22 | Day 60C | Campylobacteraceae |  | 1 |
| IL22 | Day 60C | Carnobacteriaceae | -0.019326971 | 1 |
| IL22 | Day 60C | Christensenellaceae | -0.019326971 | 1 |
| IL22 | Day 60C | Clostridiaceae | 0.525952342 | 0.015562168 |
| IL22 | Day 60C | Comamonadaceae | -0.111752886 | 1 |
| IL22 | Day 60C | Coriobacteriaceae | -0.037036309 | 1 |
| IL22 | Day 60C | Corynebacteriaceae | 0.02525133 | 1 |
| IL22 | Day 60C | Dermabacteraceae |  | 1 |
| IL22 | Day 60C | Desulfovibrionaceae | -0.185868002 | 1 |
| IL22 | Day 60C | Dietziaceae |  | 1 |
| IL22 | Day 60C | Enterobacteriaceae | 0.458738689 | 0.04777431 |
| IL22 | Day 60C | Enterococcaceae | -0.101631124 | 1 |
| IL22 | Day 60C | Erysipelotrichaceae | 0.292803144 | 0.870289101 |
| IL22 | Day 60C | Eubacteriaceae | -0.238365977 | 1 |
| IL22 | Day 60C | Fibrobacteraceae |  | 1 |
| IL22 | Day 60C | Flavobacteriaceae |  | 1 |
| IL22 | Day 60C | Fusobacteriaceae | 0.12240415 | 1 |
| IL22 | Day 60C | Gemellaceae | -0.036118433 | 1 |
| IL22 | Day 60C | Helicobacteraceae |  | 1 |
| IL22 | Day 60C | Lachnospiraceae | -0.095447336 | 1 |
| IL22 | Day 60C | Lactobacillaceae | -0.144370997 | 1 |
| IL22 | Day 60C | Leptotrichiaceae |  | 1 |
| IL22 | Day 60C | Leuconostocaceae |  | 1 |
| IL22 | Day 60C | Micrococcaceae | -0.114969729 | 1 |
| IL22 | Day 60C | mitochondria |  | 1 |
| IL22 | Day 60C | Mogibacteriaceae | -0.019326971 | 1 |
| IL22 | Day 60C | Moraxellaceae |  | 1 |
| IL22 | Day 60C | Neisseriaceae | -0.135288798 | 1 |
| IL22 | Day 60C | Odoribacteraceae | -0.019326971 | 1 |
| IL22 | Day 60C | Other | -0.305573152 | 0.870289101 |
| IL22 | Day 60C | Other | -0.026305989 | 1 |
| IL22 | Day 60C | Other | 0.247100884 | 1 |
| IL22 | Day 60C | Other |  | 1 |
| IL22 | Day 60C | Other |  | 1 |
| IL22 | Day 60C | Paenibacillaceae |  | 1 |
| IL22 | Day 60C | Paraprevotellaceae |  | 1 |
| IL22 | Day 60C | Pasteurellaceae | -0.075616345 | 1 |
| IL22 | Day 60C | Peptococcaceae |  | 1 |
| IL22 | Day 60C | Peptostreptococcaceae | 0.124559232 | 1 |
| IL22 | Day 60C | Planococcaceae | -0.133732359 | 1 |
| IL22 | Day 60C | Porphyromonadaceae | -0.164497109 | 1 |
| IL22 | Day 60C | Prevotellaceae | -0.037561192 | 1 |
| IL22 | Day 60C | Pseudomonadaceae |  | 1 |
| IL22 | Day 60C | RFP12 |  | 1 |
| IL22 | Day 60C | Rikenellaceae |  | 1 |
| IL22 | Day 60C | Ruminococcaceae | 0.015241668 | 1 |
| IL22 | Day 60C | Spirochaetaceae |  | 1 |
| IL22 | Day 60C | Staphylococcaceae | 0.248437254 | 1 |
| IL22 | Day 60C | Streptococcaceae | 0.121140912 | 1 |
| IL22 | Day 60C | Succinivibrionaceae |  | 1 |
| IL22 | Day 60C | Tissierellaceae | -0.026840246 | 1 |
| IL22 | Day 60C | Turicibacteraceae |  | 1 |
| IL22 | Day 60C | Veillonellaceae | 0.053339823 | 1 |
| IL22 | Day 60C | Verrucomicrobiaceae | -0.17394274 | 1 |
| IL22 | Day 60C | Xanthomonadaceae |  | 1 |
| IL5 | Day 6 | Actinomycetaceae | -0.082776067 | 1 |
| IL5 | Day 6 | Aerococcaceae |  | 1 |
| IL5 | Day 6 | Aeromonadaceae |  | 1 |
| IL5 | Day 6 | Alcaligenaceae | 0.224440193 | 1 |
| IL5 | Day 6 | Bacillaceae |  | 1 |
| IL5 | Day 6 | Bacteroidaceae | 0.242703374 | 1 |
| IL5 | Day 6 | Barnesiellaceae |  | 1 |
| IL5 | Day 6 | Bifidobacteriaceae | 0.046978969 | 1 |
| IL5 | Day 6 | BS11 |  | 1 |
| IL5 | Day 6 | Burkholderiaceae |  | 1 |
| IL5 | Day 6 | Campylobacteraceae |  | 1 |
| IL5 | Day 6 | Carnobacteriaceae |  | 1 |
| IL5 | Day 6 | Christensenellaceae |  | 1 |
| IL5 | Day 6 | Clostridiaceae | -0.195037981 | 1 |
| IL5 | Day 6 | Comamonadaceae | -0.159331967 | 1 |
| IL5 | Day 6 | Coriobacteriaceae | -0.15646162 | 1 |
| IL5 | Day 6 | Corynebacteriaceae | 0.075534813 | 1 |
| IL5 | Day 6 | Dermabacteraceae |  | 1 |
| IL5 | Day 6 | Desulfovibrionaceae | -0.044449332 | 1 |
| IL5 | Day 6 | Dietziaceae |  | 1 |
| IL5 | Day 6 | Enterobacteriaceae | 0.203812689 | 1 |
| IL5 | Day 6 | Enterococcaceae | -0.144133492 | 1 |
| IL5 | Day 6 | Erysipelotrichaceae | 0.139081973 | 1 |
| IL5 | Day 6 | Eubacteriaceae | 0.232924157 | 1 |
| IL5 | Day 6 | Fibrobacteraceae |  | 1 |
| IL5 | Day 6 | Flavobacteriaceae |  | 1 |
| IL5 | Day 6 | Fusobacteriaceae |  | 1 |
| IL5 | Day 6 | Gemellaceae | 0.025777506 | 1 |
| IL5 | Day 6 | Helicobacteraceae |  | 1 |
| IL5 | Day 6 | Lachnospiraceae | -0.035129873 | 1 |
| IL5 | Day 6 | Lactobacillaceae | -0.142354071 | 1 |
| IL5 | Day 6 | Leptotrichiaceae |  | 1 |
| IL5 | Day 6 | Leuconostocaceae |  | 1 |
| IL5 | Day 6 | Micrococcaceae | -0.179474767 | 1 |
| IL5 | Day 6 | mitochondria |  | 1 |
| IL5 | Day 6 | Mogibacteriaceae |  | 1 |
| IL5 | Day 6 | Moraxellaceae |  | 1 |
| IL5 | Day 6 | Neisseriaceae | 0.068257145 | 1 |
| IL5 | Day 6 | Odoribacteraceae | -0.054805684 | 1 |
| IL5 | Day 6 | Other | -0.204748723 | 1 |
| IL5 | Day 6 | Other | -0.156784602 | 1 |
| IL5 | Day 6 | Other | -0.01355161 | 1 |
| IL5 | Day 6 | Other |  | 1 |
| IL5 | Day 6 | Other |  | 1 |
| IL5 | Day 6 | Paenibacillaceae |  | 1 |
| IL5 | Day 6 | Paraprevotellaceae |  | 1 |
| IL5 | Day 6 | Pasteurellaceae | -0.090135459 | 1 |
| IL5 | Day 6 | Peptococcaceae |  | 1 |
| IL5 | Day 6 | Peptostreptococcaceae | -0.255026843 | 1 |
| IL5 | Day 6 | Planococcaceae | -0.007680281 | 1 |
| IL5 | Day 6 | Porphyromonadaceae | 0.098978269 | 1 |
| IL5 | Day 6 | Prevotellaceae |  | 1 |
| IL5 | Day 6 | Pseudomonadaceae | 0.150715631 | 1 |
| IL5 | Day 6 | RFP12 |  | 1 |
| IL5 | Day 6 | Rikenellaceae | -0.054805684 | 1 |
| IL5 | Day 6 | Ruminococcaceae | 0.236207779 | 1 |
| IL5 | Day 6 | Spirochaetaceae |  | 1 |
| IL5 | Day 6 | Staphylococcaceae | -0.111999291 | 1 |
| IL5 | Day 6 | Streptococcaceae | -0.123431435 | 1 |
| IL5 | Day 6 | Succinivibrionaceae |  | 1 |
| IL5 | Day 6 | Tissierellaceae |  | 1 |
| IL5 | Day 6 | Turicibacteraceae |  | 1 |
| IL5 | Day 6 | Veillonellaceae | -0.222988678 | 1 |
| IL5 | Day 6 | Verrucomicrobiaceae |  | 1 |
| IL5 | Day 6 | Xanthomonadaceae |  | 1 |
| IL5 | Day 40 | Actinomycetaceae | -0.272887091 | 1 |
| IL5 | Day 40 | Aerococcaceae |  | 1 |
| IL5 | Day 40 | Aeromonadaceae |  | 1 |
| IL5 | Day 40 | Alcaligenaceae | 0.105516475 | 1 |
| IL5 | Day 40 | Bacillaceae |  | 1 |
| IL5 | Day 40 | Bacteroidaceae | 0.06921511 | 1 |
| IL5 | Day 40 | Barnesiellaceae | 0.149209419 | 1 |
| IL5 | Day 40 | Bifidobacteriaceae | -0.199939595 | 1 |
| IL5 | Day 40 | BS11 |  | 1 |
| IL5 | Day 40 | Burkholderiaceae |  | 1 |
| IL5 | Day 40 | Campylobacteraceae |  | 1 |
| IL5 | Day 40 | Carnobacteriaceae |  | 1 |
| IL5 | Day 40 | Christensenellaceae |  | 1 |
| IL5 | Day 40 | Clostridiaceae | 0.274362057 | 1 |
| IL5 | Day 40 | Comamonadaceae | 0.208376081 | 1 |
| IL5 | Day 40 | Coriobacteriaceae | -0.106734667 | 1 |
| IL5 | Day 40 | Corynebacteriaceae | -0.130876342 | 1 |
| IL5 | Day 40 | Dermabacteraceae |  | 1 |
| IL5 | Day 40 | Desulfovibrionaceae | -0.215233942 | 1 |
| IL5 | Day 40 | Dietziaceae |  | 1 |
| IL5 | Day 40 | Enterobacteriaceae | 0.310225045 | 1 |
| IL5 | Day 40 | Enterococcaceae | 0.013165889 | 1 |
| IL5 | Day 40 | Erysipelotrichaceae | 0.261168342 | 1 |
| IL5 | Day 40 | Eubacteriaceae | -0.124341183 | 1 |
| IL5 | Day 40 | Fibrobacteraceae |  | 1 |
| IL5 | Day 40 | Flavobacteriaceae |  | 1 |
| IL5 | Day 40 | Fusobacteriaceae |  | 1 |
| IL5 | Day 40 | Gemellaceae | -0.072762533 | 1 |
| IL5 | Day 40 | Helicobacteraceae |  | 1 |
| IL5 | Day 40 | Lachnospiraceae | -0.016078668 | 1 |
| IL5 | Day 40 | Lactobacillaceae | 0.128351229 | 1 |
| IL5 | Day 40 | Leptotrichiaceae |  | 1 |
| IL5 | Day 40 | Leuconostocaceae |  | 1 |
| IL5 | Day 40 | Micrococcaceae | -0.107572946 | 1 |
| IL5 | Day 40 | mitochondria |  | 1 |
| IL5 | Day 40 | Mogibacteriaceae |  | 1 |
| IL5 | Day 40 | Moraxellaceae |  | 1 |
| IL5 | Day 40 | Neisseriaceae | -0.266475316 | 1 |
| IL5 | Day 40 | Odoribacteraceae | -0.174077656 | 1 |
| IL5 | Day 40 | Other | -0.174077656 | 1 |
| IL5 | Day 40 | Other | -0.162752467 | 1 |
| IL5 | Day 40 | Other | 0.183618006 | 1 |
| IL5 | Day 40 | Other | 0.244684147 | 1 |
| IL5 | Day 40 | Other | 0.248682366 | 1 |
| IL5 | Day 40 | Paenibacillaceae |  | 1 |
| IL5 | Day 40 | Paraprevotellaceae |  | 1 |
| IL5 | Day 40 | Pasteurellaceae | -0.189669192 | 1 |
| IL5 | Day 40 | Peptococcaceae |  | 1 |
| IL5 | Day 40 | Peptostreptococcaceae | 0.44737983 | 0.169989318 |
| IL5 | Day 40 | Planococcaceae | 0.117927741 | 1 |
| IL5 | Day 40 | Porphyromonadaceae | -0.017757561 | 1 |
| IL5 | Day 40 | Prevotellaceae |  | 1 |
| IL5 | Day 40 | Pseudomonadaceae |  | 1 |
| IL5 | Day 40 | RFP12 |  | 1 |
| IL5 | Day 40 | Rikenellaceae |  | 1 |
| IL5 | Day 40 | Ruminococcaceae | 0.181458262 | 1 |
| IL5 | Day 40 | Spirochaetaceae |  | 1 |
| IL5 | Day 40 | Staphylococcaceae | -0.072588569 | 1 |
| IL5 | Day 40 | Streptococcaceae | -0.046058593 | 1 |
| IL5 | Day 40 | Succinivibrionaceae |  | 1 |
| IL5 | Day 40 | Tissierellaceae | -0.212544317 | 1 |
| IL5 | Day 40 | Turicibacteraceae |  | 1 |
| IL5 | Day 40 | Veillonellaceae | 0.033444385 | 1 |
| IL5 | Day 40 | Verrucomicrobiaceae | -0.174077656 | 1 |
| IL5 | Day 40 | Xanthomonadaceae |  | 1 |
| IL5 | Day 60C | Actinomycetaceae | -0.311963625 | 0.533687479 |
| IL5 | Day 60C | Aerococcaceae |  | 1 |
| IL5 | Day 60C | Aeromonadaceae |  | 1 |
| IL5 | Day 60C | Alcaligenaceae | -0.104248772 | 1 |
| IL5 | Day 60C | Bacillaceae |  | 1 |
| IL5 | Day 60C | Bacteroidaceae | -0.189708988 | 0.93450909 |
| IL5 | Day 60C | Barnesiellaceae |  | 1 |
| IL5 | Day 60C | Bifidobacteriaceae | -0.38724319 | 0.226693112 |
| IL5 | Day 60C | BS11 |  | 1 |
| IL5 | Day 60C | Burkholderiaceae |  | 1 |
| IL5 | Day 60C | Campylobacteraceae |  | 1 |
| IL5 | Day 60C | Carnobacteriaceae | 0.006443629 | 1 |
| IL5 | Day 60C | Christensenellaceae | 0.006443629 | 1 |
| IL5 | Day 60C | Clostridiaceae | 0.369190958 | 0.226693112 |
| IL5 | Day 60C | Comamonadaceae | -0.260992573 | 0.695085845 |
| IL5 | Day 60C | Coriobacteriaceae | -0.268020313 | 0.695085845 |
| IL5 | Day 60C | Corynebacteriaceae | 0.050617693 | 1 |
| IL5 | Day 60C | Dermabacteraceae |  | 1 |
| IL5 | Day 60C | Desulfovibrionaceae | -0.232581838 | 0.724754491 |
| IL5 | Day 60C | Dietziaceae |  | 1 |
| IL5 | Day 60C | Enterobacteriaceae | 0.481452963 | 0.076362303 |
| IL5 | Day 60C | Enterococcaceae | 0.269381141 | 0.695085845 |
| IL5 | Day 60C | Erysipelotrichaceae | 0.056424042 | 1 |
| IL5 | Day 60C | Eubacteriaceae | -0.045105404 | 1 |
| IL5 | Day 60C | Fibrobacteraceae |  | 1 |
| IL5 | Day 60C | Flavobacteriaceae |  | 1 |
| IL5 | Day 60C | Fusobacteriaceae | -0.148203471 | 1 |
| IL5 | Day 60C | Gemellaceae | 0.014467627 | 1 |
| IL5 | Day 60C | Helicobacteraceae |  | 1 |
| IL5 | Day 60C | Lachnospiraceae | -0.218766722 | 0.802717886 |
| IL5 | Day 60C | Lactobacillaceae | -0.24274751 | 0.724754491 |
| IL5 | Day 60C | Leptotrichiaceae |  | 1 |
| IL5 | Day 60C | Leuconostocaceae |  | 1 |
| IL5 | Day 60C | Micrococcaceae | -0.250966791 | 0.724754491 |
| IL5 | Day 60C | mitochondria |  | 1 |
| IL5 | Day 60C | Mogibacteriaceae | 0.006443629 | 1 |
| IL5 | Day 60C | Moraxellaceae |  | 1 |
| IL5 | Day 60C | Neisseriaceae | -0.199752504 | 0.884875745 |
| IL5 | Day 60C | Odoribacteraceae | 0.006443629 | 1 |
| IL5 | Day 60C | Other | 0.209109014 | 0.844185509 |
| IL5 | Day 60C | Other | -0.157303299 | 1 |
| IL5 | Day 60C | Other | 0.032753242 | 1 |
| IL5 | Day 60C | Other |  | 1 |
| IL5 | Day 60C | Other |  | 1 |
| IL5 | Day 60C | Paenibacillaceae |  | 1 |
| IL5 | Day 60C | Paraprevotellaceae |  | 1 |
| IL5 | Day 60C | Pasteurellaceae | 0.035498089 | 1 |
| IL5 | Day 60C | Peptococcaceae |  | 1 |
| IL5 | Day 60C | Peptostreptococcaceae | 0.379799663 | 0.226693112 |
| IL5 | Day 60C | Planococcaceae | -0.124321884 | 1 |
| IL5 | Day 60C | Porphyromonadaceae | -0.376150243 | 0.226693112 |
| IL5 | Day 60C | Prevotellaceae | -0.065518541 | 1 |
| IL5 | Day 60C | Pseudomonadaceae |  | 1 |
| IL5 | Day 60C | RFP12 |  | 1 |
| IL5 | Day 60C | Rikenellaceae |  | 1 |
| IL5 | Day 60C | Ruminococcaceae | -0.285923871 | 0.690800188 |
| IL5 | Day 60C | Spirochaetaceae |  | 1 |
| IL5 | Day 60C | Staphylococcaceae | 0.089111113 | 1 |
| IL5 | Day 60C | Streptococcaceae | -0.017344086 | 1 |
| IL5 | Day 60C | Succinivibrionaceae |  | 1 |
| IL5 | Day 60C | Tissierellaceae | -0.129852696 | 1 |
| IL5 | Day 60C | Turicibacteraceae |  | 1 |
| IL5 | Day 60C | Veillonellaceae | 0.025702356 | 1 |
| IL5 | Day 60C | Verrucomicrobiaceae | 0.032218146 | 1 |
| IL5 | Day 60C | Xanthomonadaceae |  | 1 |
| IL6 | Day 6 | Actinomycetaceae | -0.197646995 | 1 |
| IL6 | Day 6 | Aerococcaceae |  | 1 |
| IL6 | Day 6 | Aeromonadaceae |  | 1 |
| IL6 | Day 6 | Alcaligenaceae | 0.236186706 | 1 |
| IL6 | Day 6 | Bacillaceae |  | 1 |
| IL6 | Day 6 | Bacteroidaceae | 0.079798185 | 1 |
| IL6 | Day 6 | Barnesiellaceae |  | 1 |
| IL6 | Day 6 | Bifidobacteriaceae | 0.082122262 | 1 |
| IL6 | Day 6 | BS11 |  | 1 |
| IL6 | Day 6 | Burkholderiaceae |  | 1 |
| IL6 | Day 6 | Campylobacteraceae |  | 1 |
| IL6 | Day 6 | Carnobacteriaceae |  | 1 |
| IL6 | Day 6 | Christensenellaceae |  | 1 |
| IL6 | Day 6 | Clostridiaceae | -0.088967419 | 1 |
| IL6 | Day 6 | Comamonadaceae | -0.043132207 | 1 |
| IL6 | Day 6 | Coriobacteriaceae | -0.115655983 | 1 |
| IL6 | Day 6 | Corynebacteriaceae | 0.02110515 | 1 |
| IL6 | Day 6 | Dermabacteraceae |  | 1 |
| IL6 | Day 6 | Desulfovibrionaceae | -0.010013393 | 1 |
| IL6 | Day 6 | Dietziaceae |  | 1 |
| IL6 | Day 6 | Enterobacteriaceae | 0.263077332 | 1 |
| IL6 | Day 6 | Enterococcaceae | -0.117820239 | 1 |
| IL6 | Day 6 | Erysipelotrichaceae | 0.209405166 | 1 |
| IL6 | Day 6 | Eubacteriaceae | 0.188900467 | 1 |
| IL6 | Day 6 | Fibrobacteraceae |  | 1 |
| IL6 | Day 6 | Flavobacteriaceae |  | 1 |
| IL6 | Day 6 | Fusobacteriaceae |  | 1 |
| IL6 | Day 6 | Gemellaceae | 0.018081375 | 1 |
| IL6 | Day 6 | Helicobacteraceae |  | 1 |
| IL6 | Day 6 | Lachnospiraceae | -0.174677449 | 1 |
| IL6 | Day 6 | Lactobacillaceae | 0.128017372 | 1 |
| IL6 | Day 6 | Leptotrichiaceae |  | 1 |
| IL6 | Day 6 | Leuconostocaceae |  | 1 |
| IL6 | Day 6 | Micrococcaceae | -0.245442373 | 1 |
| IL6 | Day 6 | mitochondria |  | 1 |
| IL6 | Day 6 | Mogibacteriaceae |  | 1 |
| IL6 | Day 6 | Moraxellaceae |  | 1 |
| IL6 | Day 6 | Neisseriaceae | 0.05179652 | 1 |
| IL6 | Day 6 | Odoribacteraceae | -0.138042649 | 1 |
| IL6 | Day 6 | Other | -0.23790368 | 1 |
| IL6 | Day 6 | Other | -0.159588262 | 1 |
| IL6 | Day 6 | Other | 0.038424941 | 1 |
| IL6 | Day 6 | Other |  | 1 |
| IL6 | Day 6 | Other |  | 1 |
| IL6 | Day 6 | Paenibacillaceae |  | 1 |
| IL6 | Day 6 | Paraprevotellaceae |  | 1 |
| IL6 | Day 6 | Pasteurellaceae | -0.012758434 | 1 |
| IL6 | Day 6 | Peptococcaceae |  | 1 |
| IL6 | Day 6 | Peptostreptococcaceae | -0.043132207 | 1 |
| IL6 | Day 6 | Planococcaceae | 0.066251363 | 1 |
| IL6 | Day 6 | Porphyromonadaceae | -0.106461064 | 1 |
| IL6 | Day 6 | Prevotellaceae |  | 1 |
| IL6 | Day 6 | Pseudomonadaceae | 0.203431272 | 1 |
| IL6 | Day 6 | RFP12 |  | 1 |
| IL6 | Day 6 | Rikenellaceae | -0.138042649 | 1 |
| IL6 | Day 6 | Ruminococcaceae | 0.048179802 | 1 |
| IL6 | Day 6 | Spirochaetaceae |  | 1 |
| IL6 | Day 6 | Staphylococcaceae | 0.032110089 | 1 |
| IL6 | Day 6 | Streptococcaceae | -0.086100503 | 1 |
| IL6 | Day 6 | Succinivibrionaceae |  | 1 |
| IL6 | Day 6 | Tissierellaceae |  | 1 |
| IL6 | Day 6 | Turicibacteraceae |  | 1 |
| IL6 | Day 6 | Veillonellaceae | -0.134477731 | 1 |
| IL6 | Day 6 | Verrucomicrobiaceae |  | 1 |
| IL6 | Day 6 | Xanthomonadaceae |  | 1 |
| IL6 | Day 40 | Actinomycetaceae | -0.192615044 | 1 |
| IL6 | Day 40 | Aerococcaceae |  | 1 |
| IL6 | Day 40 | Aeromonadaceae |  | 1 |
| IL6 | Day 40 | Alcaligenaceae | 0.102227837 | 1 |
| IL6 | Day 40 | Bacillaceae |  | 1 |
| IL6 | Day 40 | Bacteroidaceae | 0.022558821 | 1 |
| IL6 | Day 40 | Barnesiellaceae | 0.112148659 | 1 |
| IL6 | Day 40 | Bifidobacteriaceae | -0.330900092 | 0.541260262 |
| IL6 | Day 40 | BS11 |  | 1 |
| IL6 | Day 40 | Burkholderiaceae |  | 1 |
| IL6 | Day 40 | Campylobacteraceae |  | 1 |
| IL6 | Day 40 | Carnobacteriaceae |  | 1 |
| IL6 | Day 40 | Christensenellaceae |  | 1 |
| IL6 | Day 40 | Clostridiaceae | 0.262196367 | 0.933586388 |
| IL6 | Day 40 | Comamonadaceae | 0.344160227 | 0.541260262 |
| IL6 | Day 40 | Coriobacteriaceae | 0.053274444 | 1 |
| IL6 | Day 40 | Corynebacteriaceae | -0.147196971 | 1 |
| IL6 | Day 40 | Dermabacteraceae |  | 1 |
| IL6 | Day 40 | Desulfovibrionaceae | -0.086170711 | 1 |
| IL6 | Day 40 | Dietziaceae |  | 1 |
| IL6 | Day 40 | Enterobacteriaceae | 0.275097991 | 0.933586388 |
| IL6 | Day 40 | Enterococcaceae | -0.120720379 | 1 |
| IL6 | Day 40 | Erysipelotrichaceae | 0.171934632 | 1 |
| IL6 | Day 40 | Eubacteriaceae | -0.224297319 | 1 |
| IL6 | Day 40 | Fibrobacteraceae |  | 1 |
| IL6 | Day 40 | Flavobacteriaceae |  | 1 |
| IL6 | Day 40 | Fusobacteriaceae |  | 1 |
| IL6 | Day 40 | Gemellaceae | -0.050780457 | 1 |
| IL6 | Day 40 | Helicobacteraceae |  | 1 |
| IL6 | Day 40 | Lachnospiraceae | 0.263075586 | 0.933586388 |
| IL6 | Day 40 | Lactobacillaceae | -0.047813032 | 1 |
| IL6 | Day 40 | Leptotrichiaceae |  | 1 |
| IL6 | Day 40 | Leuconostocaceae |  | 1 |
| IL6 | Day 40 | Micrococcaceae | 0.033956259 | 1 |
| IL6 | Day 40 | mitochondria |  | 1 |
| IL6 | Day 40 | Mogibacteriaceae |  | 1 |
| IL6 | Day 40 | Moraxellaceae |  | 1 |
| IL6 | Day 40 | Neisseriaceae | -0.238852717 | 1 |
| IL6 | Day 40 | Odoribacteraceae | -0.112148659 | 1 |
| IL6 | Day 40 | Other | 0.353035067 | 0.541260262 |
| IL6 | Day 40 | Other | -0.112148659 | 1 |
| IL6 | Day 40 | Other | 0.012235447 | 1 |
| IL6 | Day 40 | Other | 0.196058454 | 1 |
| IL6 | Day 40 | Other | 0.224297319 | 1 |
| IL6 | Day 40 | Paenibacillaceae |  | 1 |
| IL6 | Day 40 | Paraprevotellaceae |  | 1 |
| IL6 | Day 40 | Pasteurellaceae | -0.005199836 | 1 |
| IL6 | Day 40 | Peptococcaceae |  | 1 |
| IL6 | Day 40 | Peptostreptococcaceae | 0.480495952 | 0.067688745 |
| IL6 | Day 40 | Planococcaceae | -0.074641474 | 1 |
| IL6 | Day 40 | Porphyromonadaceae | -0.019518081 | 1 |
| IL6 | Day 40 | Prevotellaceae |  | 1 |
| IL6 | Day 40 | Pseudomonadaceae |  | 1 |
| IL6 | Day 40 | RFP12 |  | 1 |
| IL6 | Day 40 | Rikenellaceae |  | 1 |
| IL6 | Day 40 | Ruminococcaceae | 0.161756086 | 1 |
| IL6 | Day 40 | Spirochaetaceae |  | 1 |
| IL6 | Day 40 | Staphylococcaceae | -0.087279196 | 1 |
| IL6 | Day 40 | Streptococcaceae | -0.087700253 | 1 |
| IL6 | Day 40 | Succinivibrionaceae |  | 1 |
| IL6 | Day 40 | Tissierellaceae | -0.183638104 | 1 |
| IL6 | Day 40 | Turicibacteraceae |  | 1 |
| IL6 | Day 40 | Veillonellaceae | -0.025023697 | 1 |
| IL6 | Day 40 | Verrucomicrobiaceae | -0.112148659 | 1 |
| IL6 | Day 40 | Xanthomonadaceae |  | 1 |
| IL6 | Day 60C | Actinomycetaceae | -0.287446628 | 0.818000517 |
| IL6 | Day 60C | Aerococcaceae |  | 1 |
| IL6 | Day 60C | Aeromonadaceae |  | 1 |
| IL6 | Day 60C | Alcaligenaceae | 0.126127414 | 1 |
| IL6 | Day 60C | Bacillaceae |  | 1 |
| IL6 | Day 60C | Bacteroidaceae | 0.051678487 | 1 |
| IL6 | Day 60C | Barnesiellaceae |  | 1 |
| IL6 | Day 60C | Bifidobacteriaceae | -0.481257861 | 0.037387514 |
| IL6 | Day 60C | BS11 |  | 1 |
| IL6 | Day 60C | Burkholderiaceae |  | 1 |
| IL6 | Day 60C | Campylobacteraceae |  | 1 |
| IL6 | Day 60C | Carnobacteriaceae | -0.109541696 | 1 |
| IL6 | Day 60C | Christensenellaceae | -0.109541696 | 1 |
| IL6 | Day 60C | Clostridiaceae | 0.581142327 | 0.002450084 |
| IL6 | Day 60C | Comamonadaceae | -0.101015215 | 1 |
| IL6 | Day 60C | Coriobacteriaceae | 0.066643835 | 1 |
| IL6 | Day 60C | Corynebacteriaceae | -0.085620404 | 1 |
| IL6 | Day 60C | Dermabacteraceae |  | 1 |
| IL6 | Day 60C | Desulfovibrionaceae | -0.193629541 | 1 |
| IL6 | Day 60C | Dietziaceae |  | 1 |
| IL6 | Day 60C | Enterobacteriaceae | 0.467790985 | 0.037387514 |
| IL6 | Day 60C | Enterococcaceae | 0.003352953 | 1 |
| IL6 | Day 60C | Erysipelotrichaceae | 0.370228519 | 0.277570837 |
| IL6 | Day 60C | Eubacteriaceae | -0.238414279 | 1 |
| IL6 | Day 60C | Fibrobacteraceae |  | 1 |
| IL6 | Day 60C | Flavobacteriaceae |  | 1 |
| IL6 | Day 60C | Fusobacteriaceae | 0.045105404 | 1 |
| IL6 | Day 60C | Gemellaceae | -0.024776894 | 1 |
| IL6 | Day 60C | Helicobacteraceae |  | 1 |
| IL6 | Day 60C | Lachnospiraceae | -0.040065866 | 1 |
| IL6 | Day 60C | Lactobacillaceae | -0.027140061 | 1 |
| IL6 | Day 60C | Leptotrichiaceae |  | 1 |
| IL6 | Day 60C | Leuconostocaceae |  | 1 |
| IL6 | Day 60C | Micrococcaceae | -0.195960949 | 1 |
| IL6 | Day 60C | mitochondria |  | 1 |
| IL6 | Day 60C | Mogibacteriaceae | -0.109541696 | 1 |
| IL6 | Day 60C | Moraxellaceae |  | 1 |
| IL6 | Day 60C | Neisseriaceae | -0.135316213 | 1 |
| IL6 | Day 60C | Odoribacteraceae | -0.109541696 | 1 |
| IL6 | Day 60C | Other | 0.274567111 | 0.818000517 |
| IL6 | Day 60C | Other | -0.124555304 | 1 |
| IL6 | Day 60C | Other | -0.101785572 | 1 |
| IL6 | Day 60C | Other |  | 1 |
| IL6 | Day 60C | Other |  | 1 |
| IL6 | Day 60C | Paenibacillaceae |  | 1 |
| IL6 | Day 60C | Paraprevotellaceae |  | 1 |
| IL6 | Day 60C | Pasteurellaceae | -0.217624024 | 1 |
| IL6 | Day 60C | Peptococcaceae |  | 1 |
| IL6 | Day 60C | Peptostreptococcaceae | 0.18647893 | 1 |
| IL6 | Day 60C | Planococcaceae | -0.22559431 | 1 |
| IL6 | Day 60C | Porphyromonadaceae | -0.108407155 | 1 |
| IL6 | Day 60C | Prevotellaceae | -0.13194454 | 1 |
| IL6 | Day 60C | Pseudomonadaceae |  | 1 |
| IL6 | Day 60C | RFP12 |  | 1 |
| IL6 | Day 60C | Rikenellaceae |  | 1 |
| IL6 | Day 60C | Ruminococcaceae | 0.032409222 | 1 |
| IL6 | Day 60C | Spirochaetaceae |  | 1 |
| IL6 | Day 60C | Staphylococcaceae | 0.122497318 | 1 |
| IL6 | Day 60C | Streptococcaceae | 0.156015726 | 1 |
| IL6 | Day 60C | Succinivibrionaceae |  | 1 |
| IL6 | Day 60C | Tissierellaceae | -0.019513383 | 1 |
| IL6 | Day 60C | Turicibacteraceae |  | 1 |
| IL6 | Day 60C | Veillonellaceae | 0.200835128 | 1 |
| IL6 | Day 60C | Verrucomicrobiaceae | -0.161090729 | 1 |
| IL6 | Day 60C | Xanthomonadaceae |  | 1 |
| IL8 | Day 6 | Actinomycetaceae | 0.084002165 | 1 |
| IL8 | Day 6 | Aerococcaceae |  | 1 |
| IL8 | Day 6 | Aeromonadaceae |  | 1 |
| IL8 | Day 6 | Alcaligenaceae | -0.024022267 | 1 |
| IL8 | Day 6 | Bacillaceae |  | 1 |
| IL8 | Day 6 | Bacteroidaceae | 0.136596518 | 1 |
| IL8 | Day 6 | Barnesiellaceae |  | 1 |
| IL8 | Day 6 | Bifidobacteriaceae | -0.028659785 | 1 |
| IL8 | Day 6 | BS11 |  | 1 |
| IL8 | Day 6 | Burkholderiaceae |  | 1 |
| IL8 | Day 6 | Campylobacteraceae |  | 1 |
| IL8 | Day 6 | Carnobacteriaceae |  | 1 |
| IL8 | Day 6 | Christensenellaceae |  | 1 |
| IL8 | Day 6 | Clostridiaceae | 0.265216998 | 1 |
| IL8 | Day 6 | Comamonadaceae | 0.188071515 | 1 |
| IL8 | Day 6 | Coriobacteriaceae | -0.011965437 | 1 |
| IL8 | Day 6 | Corynebacteriaceae | -0.145078502 | 1 |
| IL8 | Day 6 | Dermabacteraceae |  | 1 |
| IL8 | Day 6 | Desulfovibrionaceae | -0.119576422 | 1 |
| IL8 | Day 6 | Dietziaceae |  | 1 |
| IL8 | Day 6 | Enterobacteriaceae | -0.098783765 | 1 |
| IL8 | Day 6 | Enterococcaceae | -0.043200973 | 1 |
| IL8 | Day 6 | Erysipelotrichaceae | 0.161960123 | 1 |
| IL8 | Day 6 | Eubacteriaceae | -0.120272797 | 1 |
| IL8 | Day 6 | Fibrobacteraceae |  | 1 |
| IL8 | Day 6 | Flavobacteriaceae |  | 1 |
| IL8 | Day 6 | Fusobacteriaceae |  | 1 |
| IL8 | Day 6 | Gemellaceae | -0.113183202 | 1 |
| IL8 | Day 6 | Helicobacteraceae |  | 1 |
| IL8 | Day 6 | Lachnospiraceae | 0.176655303 | 1 |
| IL8 | Day 6 | Lactobacillaceae | -0.068855617 | 1 |
| IL8 | Day 6 | Leptotrichiaceae |  | 1 |
| IL8 | Day 6 | Leuconostocaceae |  | 1 |
| IL8 | Day 6 | Micrococcaceae | -0.042862678 | 1 |
| IL8 | Day 6 | mitochondria |  | 1 |
| IL8 | Day 6 | Mogibacteriaceae |  | 1 |
| IL8 | Day 6 | Moraxellaceae |  | 1 |
| IL8 | Day 6 | Neisseriaceae | -0.036135879 | 1 |
| IL8 | Day 6 | Odoribacteraceae | -0.147000086 | 1 |
| IL8 | Day 6 | Other | -0.318413889 | 1 |
| IL8 | Day 6 | Other | -0.041693696 | 1 |
| IL8 | Day 6 | Other | 0.142639142 | 1 |
| IL8 | Day 6 | Other |  | 1 |
| IL8 | Day 6 | Other |  | 1 |
| IL8 | Day 6 | Paenibacillaceae |  | 1 |
| IL8 | Day 6 | Paraprevotellaceae |  | 1 |
| IL8 | Day 6 | Pasteurellaceae | 0.013022558 | 1 |
| IL8 | Day 6 | Peptococcaceae |  | 1 |
| IL8 | Day 6 | Peptostreptococcaceae | 0.104069349 | 1 |
| IL8 | Day 6 | Planococcaceae | 0.040303375 | 1 |
| IL8 | Day 6 | Porphyromonadaceae | 0.11558206 | 1 |
| IL8 | Day 6 | Prevotellaceae |  | 1 |
| IL8 | Day 6 | Pseudomonadaceae | 0.026727288 | 1 |
| IL8 | Day 6 | RFP12 |  | 1 |
| IL8 | Day 6 | Rikenellaceae | -0.147000086 | 1 |
| IL8 | Day 6 | Ruminococcaceae | 0.167673237 | 1 |
| IL8 | Day 6 | Spirochaetaceae |  | 1 |
| IL8 | Day 6 | Staphylococcaceae | 0.158369267 | 1 |
| IL8 | Day 6 | Streptococcaceae | -0.248529988 | 1 |
| IL8 | Day 6 | Succinivibrionaceae |  | 1 |
| IL8 | Day 6 | Tissierellaceae |  | 1 |
| IL8 | Day 6 | Turicibacteraceae |  | 1 |
| IL8 | Day 6 | Veillonellaceae | -0.075106911 | 1 |
| IL8 | Day 6 | Verrucomicrobiaceae |  | 1 |
| IL8 | Day 6 | Xanthomonadaceae |  | 1 |
| IL8 | Day 40 | Actinomycetaceae | 0.031163032 | 1 |
| IL8 | Day 40 | Aerococcaceae |  | 1 |
| IL8 | Day 40 | Aeromonadaceae |  | 1 |
| IL8 | Day 40 | Alcaligenaceae | -0.039600007 | 1 |
| IL8 | Day 40 | Bacillaceae |  | 1 |
| IL8 | Day 40 | Bacteroidaceae | 0.199682529 | 1 |
| IL8 | Day 40 | Barnesiellaceae | 0.198945893 | 1 |
| IL8 | Day 40 | Bifidobacteriaceae | -0.526427061 | 0.014075633 |
| IL8 | Day 40 | BS11 |  | 1 |
| IL8 | Day 40 | Burkholderiaceae |  | 1 |
| IL8 | Day 40 | Campylobacteraceae |  | 1 |
| IL8 | Day 40 | Carnobacteriaceae |  | 1 |
| IL8 | Day 40 | Christensenellaceae |  | 1 |
| IL8 | Day 40 | Clostridiaceae | 0.508912296 | 0.014075633 |
| IL8 | Day 40 | Comamonadaceae | 0.150427359 | 1 |
| IL8 | Day 40 | Coriobacteriaceae | 0.010216626 | 1 |
| IL8 | Day 40 | Corynebacteriaceae | -0.278705577 | 0.732144581 |
| IL8 | Day 40 | Dermabacteraceae |  | 1 |
| IL8 | Day 40 | Desulfovibrionaceae | 0.030922079 | 1 |
| IL8 | Day 40 | Dietziaceae |  | 1 |
| IL8 | Day 40 | Enterobacteriaceae | 0.476514127 | 0.025344184 |
| IL8 | Day 40 | Enterococcaceae | 0.094431205 | 1 |
| IL8 | Day 40 | Erysipelotrichaceae | 0.295035294 | 0.662649798 |
| IL8 | Day 40 | Eubacteriaceae | -0.062170591 | 1 |
| IL8 | Day 40 | Fibrobacteraceae |  | 1 |
| IL8 | Day 40 | Flavobacteriaceae |  | 1 |
| IL8 | Day 40 | Fusobacteriaceae |  | 1 |
| IL8 | Day 40 | Gemellaceae | 0.238667478 | 1 |
| IL8 | Day 40 | Helicobacteraceae |  | 1 |
| IL8 | Day 40 | Lachnospiraceae | 0.139785967 | 1 |
| IL8 | Day 40 | Lactobacillaceae | -0.030039649 | 1 |
| IL8 | Day 40 | Leptotrichiaceae |  | 1 |
| IL8 | Day 40 | Leuconostocaceae |  | 1 |
| IL8 | Day 40 | Micrococcaceae | 0.121315746 | 1 |
| IL8 | Day 40 | mitochondria |  | 1 |
| IL8 | Day 40 | Mogibacteriaceae |  | 1 |
| IL8 | Day 40 | Moraxellaceae |  | 1 |
| IL8 | Day 40 | Neisseriaceae | -0.076549586 | 1 |
| IL8 | Day 40 | Odoribacteraceae | 0.049736473 | 1 |
| IL8 | Day 40 | Other | 0.376454694 | 0.179268052 |
| IL8 | Day 40 | Other | -0.124341183 | 1 |
| IL8 | Day 40 | Other | 0.049736473 | 1 |
| IL8 | Day 40 | Other | 0.1668301 | 1 |
| IL8 | Day 40 | Other | 0.218595779 | 1 |
| IL8 | Day 40 | Paenibacillaceae |  | 1 |
| IL8 | Day 40 | Paraprevotellaceae |  | 1 |
| IL8 | Day 40 | Pasteurellaceae | 0.049462453 | 1 |
| IL8 | Day 40 | Peptococcaceae |  | 1 |
| IL8 | Day 40 | Peptostreptococcaceae | 0.410787419 | 0.105153447 |
| IL8 | Day 40 | Planococcaceae | 0.171719342 | 1 |
| IL8 | Day 40 | Porphyromonadaceae | -0.057664338 | 1 |
| IL8 | Day 40 | Prevotellaceae |  | 1 |
| IL8 | Day 40 | Pseudomonadaceae |  | 1 |
| IL8 | Day 40 | RFP12 |  | 1 |
| IL8 | Day 40 | Rikenellaceae |  | 1 |
| IL8 | Day 40 | Ruminococcaceae | 0.234843082 | 1 |
| IL8 | Day 40 | Spirochaetaceae |  | 1 |
| IL8 | Day 40 | Staphylococcaceae | 0.223204185 | 1 |
| IL8 | Day 40 | Streptococcaceae | 0.005285412 | 1 |
| IL8 | Day 40 | Succinivibrionaceae |  | 1 |
| IL8 | Day 40 | Tissierellaceae | -0.139289782 | 1 |
| IL8 | Day 40 | Turicibacteraceae |  | 1 |
| IL8 | Day 40 | Veillonellaceae | 0.109261746 | 1 |
| IL8 | Day 40 | Verrucomicrobiaceae | 0.049736473 | 1 |
| IL8 | Day 40 | Xanthomonadaceae |  | 1 |
| IL8 | Day 60C | Actinomycetaceae | -0.013252087 | 1 |
| IL8 | Day 60C | Aerococcaceae |  | 1 |
| IL8 | Day 60C | Aeromonadaceae |  | 1 |
| IL8 | Day 60C | Alcaligenaceae | 0.013124526 | 1 |
| IL8 | Day 60C | Bacillaceae |  | 1 |
| IL8 | Day 60C | Bacteroidaceae | 0.113039337 | 1 |
| IL8 | Day 60C | Barnesiellaceae |  | 1 |
| IL8 | Day 60C | Bifidobacteriaceae | -0.483024066 | 0.036487725 |
| IL8 | Day 60C | BS11 |  | 1 |
| IL8 | Day 60C | Burkholderiaceae |  | 1 |
| IL8 | Day 60C | Campylobacteraceae |  | 1 |
| IL8 | Day 60C | Carnobacteriaceae | 0.096634856 | 1 |
| IL8 | Day 60C | Christensenellaceae | 0.096634856 | 1 |
| IL8 | Day 60C | Clostridiaceae | 0.582015394 | 0.002363426 |
| IL8 | Day 60C | Comamonadaceae | -0.18815761 | 1 |
| IL8 | Day 60C | Coriobacteriaceae | 0.035110071 | 1 |
| IL8 | Day 60C | Corynebacteriaceae | -0.013097163 | 1 |
| IL8 | Day 60C | Dermabacteraceae |  | 1 |
| IL8 | Day 60C | Desulfovibrionaceae | -0.24604876 | 0.964071715 |
| IL8 | Day 60C | Dietziaceae |  | 1 |
| IL8 | Day 60C | Enterobacteriaceae | 0.448686807 | 0.062186821 |
| IL8 | Day 60C | Enterococcaceae | 0.096071255 | 1 |
| IL8 | Day 60C | Erysipelotrichaceae | 0.262752581 | 0.964071715 |
| IL8 | Day 60C | Eubacteriaceae | -0.045096266 | 1 |
| IL8 | Day 60C | Fibrobacteraceae |  | 1 |
| IL8 | Day 60C | Flavobacteriaceae |  | 1 |
| IL8 | Day 60C | Fusobacteriaceae | -0.135288798 | 1 |
| IL8 | Day 60C | Gemellaceae | -0.07284317 | 1 |
| IL8 | Day 60C | Helicobacteraceae |  | 1 |
| IL8 | Day 60C | Lachnospiraceae | -0.161028607 | 1 |
| IL8 | Day 60C | Lactobacillaceae | -0.005578185 | 1 |
| IL8 | Day 60C | Leptotrichiaceae |  | 1 |
| IL8 | Day 60C | Leuconostocaceae |  | 1 |
| IL8 | Day 60C | Micrococcaceae | -0.098256068 | 1 |
| IL8 | Day 60C | mitochondria |  | 1 |
| IL8 | Day 60C | Mogibacteriaceae | 0.096634856 | 1 |
| IL8 | Day 60C | Moraxellaceae |  | 1 |
| IL8 | Day 60C | Neisseriaceae | -0.006442324 | 1 |
| IL8 | Day 60C | Odoribacteraceae | 0.096634856 | 1 |
| IL8 | Day 60C | Other | 0.244667991 | 0.964071715 |
| IL8 | Day 60C | Other | -0.109742878 | 1 |
| IL8 | Day 60C | Other | 0.052499077 | 1 |
| IL8 | Day 60C | Other |  | 1 |
| IL8 | Day 60C | Other |  | 1 |
| IL8 | Day 60C | Paenibacillaceae |  | 1 |
| IL8 | Day 60C | Paraprevotellaceae |  | 1 |
| IL8 | Day 60C | Pasteurellaceae | -0.009187792 | 1 |
| IL8 | Day 60C | Peptococcaceae |  | 1 |
| IL8 | Day 60C | Peptostreptococcaceae | 0.175623745 | 1 |
| IL8 | Day 60C | Planococcaceae | -0.3082921 | 0.850259363 |
| IL8 | Day 60C | Porphyromonadaceae | -0.287785265 | 0.940124694 |
| IL8 | Day 60C | Prevotellaceae | -0.168934635 | 1 |
| IL8 | Day 60C | Pseudomonadaceae |  | 1 |
| IL8 | Day 60C | RFP12 |  | 1 |
| IL8 | Day 60C | Rikenellaceae |  | 1 |
| IL8 | Day 60C | Ruminococcaceae | -0.073498708 | 1 |
| IL8 | Day 60C | Spirochaetaceae |  | 1 |
| IL8 | Day 60C | Staphylococcaceae | 0.213189863 | 1 |
| IL8 | Day 60C | Streptococcaceae | 0.072360425 | 1 |
| IL8 | Day 60C | Succinivibrionaceae |  | 1 |
| IL8 | Day 60C | Tissierellaceae | 0.132782363 | 1 |
| IL8 | Day 60C | Turicibacteraceae |  | 1 |
| IL8 | Day 60C | Veillonellaceae | -0.033884568 | 1 |
| IL8 | Day 60C | Verrucomicrobiaceae | -0.264135272 | 0.964071715 |
| IL8 | Day 60C | Xanthomonadaceae |  | 1 |
| TNFa | Day 6 | Actinomycetaceae | -0.251208999 | 1 |
| TNFa | Day 6 | Aerococcaceae |  | 1 |
| TNFa | Day 6 | Aeromonadaceae |  | 1 |
| TNFa | Day 6 | Alcaligenaceae | 0.182126382 | 1 |
| TNFa | Day 6 | Bacillaceae |  | 1 |
| TNFa | Day 6 | Bacteroidaceae | 0.073831206 | 1 |
| TNFa | Day 6 | Barnesiellaceae |  | 1 |
| TNFa | Day 6 | Bifidobacteriaceae | 0.022703526 | 1 |
| TNFa | Day 6 | BS11 |  | 1 |
| TNFa | Day 6 | Burkholderiaceae |  | 1 |
| TNFa | Day 6 | Campylobacteraceae |  | 1 |
| TNFa | Day 6 | Carnobacteriaceae |  | 1 |
| TNFa | Day 6 | Christensenellaceae |  | 1 |
| TNFa | Day 6 | Clostridiaceae | -0.246617035 | 1 |
| TNFa | Day 6 | Comamonadaceae | -0.092624547 | 1 |
| TNFa | Day 6 | Coriobacteriaceae | -0.171173593 | 1 |
| TNFa | Day 6 | Corynebacteriaceae | 0.194849883 | 1 |
| TNFa | Day 6 | Dermabacteraceae |  | 1 |
| TNFa | Day 6 | Desulfovibrionaceae | -0.1227043 | 1 |
| TNFa | Day 6 | Dietziaceae |  | 1 |
| TNFa | Day 6 | Enterobacteriaceae | 0.337703617 | 0.552091115 |
| TNFa | Day 6 | Enterococcaceae | -0.358329817 | 0.552091115 |
| TNFa | Day 6 | Erysipelotrichaceae | 0.161816798 | 1 |
| TNFa | Day 6 | Eubacteriaceae | 0.187540845 | 1 |
| TNFa | Day 6 | Fibrobacteraceae |  | 1 |
| TNFa | Day 6 | Flavobacteriaceae |  | 1 |
| TNFa | Day 6 | Fusobacteriaceae |  | 1 |
| TNFa | Day 6 | Gemellaceae | 0.035195029 | 1 |
| TNFa | Day 6 | Helicobacteraceae |  | 1 |
| TNFa | Day 6 | Lachnospiraceae | -0.270452928 | 1 |
| TNFa | Day 6 | Lactobacillaceae | 0.016474188 | 1 |
| TNFa | Day 6 | Leptotrichiaceae |  | 1 |
| TNFa | Day 6 | Leuconostocaceae |  | 1 |
| TNFa | Day 6 | Micrococcaceae | -0.17005885 | 1 |
| TNFa | Day 6 | mitochondria |  | 1 |
| TNFa | Day 6 | Mogibacteriaceae |  | 1 |
| TNFa | Day 6 | Moraxellaceae |  | 1 |
| TNFa | Day 6 | Neisseriaceae | 0.118874114 | 1 |
| TNFa | Day 6 | Odoribacteraceae | -0.107166197 | 1 |
| TNFa | Day 6 | Other | -0.371653602 | 0.552091115 |
| TNFa | Day 6 | Other | -0.159911761 | 1 |
| TNFa | Day 6 | Other | 0.041404081 | 1 |
| TNFa | Day 6 | Other |  | 1 |
| TNFa | Day 6 | Other |  | 1 |
| TNFa | Day 6 | Paenibacillaceae |  | 1 |
| TNFa | Day 6 | Paraprevotellaceae |  | 1 |
| TNFa | Day 6 | Pasteurellaceae | -0.042057514 | 1 |
| TNFa | Day 6 | Peptococcaceae |  | 1 |
| TNFa | Day 6 | Peptostreptococcaceae | -0.139404621 | 1 |
| TNFa | Day 6 | Planococcaceae | 0.000951839 | 1 |
| TNFa | Day 6 | Porphyromonadaceae | -0.114960998 | 1 |
| TNFa | Day 6 | Prevotellaceae |  | 1 |
| TNFa | Day 6 | Pseudomonadaceae | 0.080374648 | 1 |
| TNFa | Day 6 | RFP12 |  | 1 |
| TNFa | Day 6 | Rikenellaceae | -0.107166197 | 1 |
| TNFa | Day 6 | Ruminococcaceae | 0.05487121 | 1 |
| TNFa | Day 6 | Spirochaetaceae |  | 1 |
| TNFa | Day 6 | Staphylococcaceae | -0.032134221 | 1 |
| TNFa | Day 6 | Streptococcaceae | 0.011351763 | 1 |
| TNFa | Day 6 | Succinivibrionaceae |  | 1 |
| TNFa | Day 6 | Tissierellaceae |  | 1 |
| TNFa | Day 6 | Turicibacteraceae |  | 1 |
| TNFa | Day 6 | Veillonellaceae | -0.11626214 | 1 |
| TNFa | Day 6 | Verrucomicrobiaceae |  | 1 |
| TNFa | Day 6 | Xanthomonadaceae |  | 1 |
| TNFa | Day 40 | Actinomycetaceae | -0.092983749 | 1 |
| TNFa | Day 40 | Aerococcaceae |  | 1 |
| TNFa | Day 40 | Aeromonadaceae |  | 1 |
| TNFa | Day 40 | Alcaligenaceae | 0.076443052 | 1 |
| TNFa | Day 40 | Bacillaceae |  | 1 |
| TNFa | Day 40 | Bacteroidaceae | 0.099228742 | 1 |
| TNFa | Day 40 | Barnesiellaceae | 0.186511774 | 1 |
| TNFa | Day 40 | Bifidobacteriaceae | -0.47372395 | 0.027469347 |
| TNFa | Day 40 | BS11 |  | 1 |
| TNFa | Day 40 | Burkholderiaceae |  | 1 |
| TNFa | Day 40 | Campylobacteraceae |  | 1 |
| TNFa | Day 40 | Carnobacteriaceae |  | 1 |
| TNFa | Day 40 | Christensenellaceae |  | 1 |
| TNFa | Day 40 | Clostridiaceae | 0.516164836 | 0.021525152 |
| TNFa | Day 40 | Comamonadaceae | 0.258119673 | 0.865652611 |
| TNFa | Day 40 | Coriobacteriaceae | -0.002574922 | 1 |
| TNFa | Day 40 | Corynebacteriaceae | -0.096699307 | 1 |
| TNFa | Day 40 | Dermabacteraceae |  | 1 |
| TNFa | Day 40 | Desulfovibrionaceae | -0.178344445 | 1 |
| TNFa | Day 40 | Dietziaceae |  | 1 |
| TNFa | Day 40 | Enterobacteriaceae | 0.451140316 | 0.038473068 |
| TNFa | Day 40 | Enterococcaceae | 0.120308987 | 1 |
| TNFa | Day 40 | Erysipelotrichaceae | 0.154153021 | 1 |
| TNFa | Day 40 | Eubacteriaceae | -0.174077656 | 1 |
| TNFa | Day 40 | Fibrobacteraceae |  | 1 |
| TNFa | Day 40 | Flavobacteriaceae |  | 1 |
| TNFa | Day 40 | Fusobacteriaceae |  | 1 |
| TNFa | Day 40 | Gemellaceae | 0.067508346 | 1 |
| TNFa | Day 40 | Helicobacteraceae |  | 1 |
| TNFa | Day 40 | Lachnospiraceae | 0.128301205 | 1 |
| TNFa | Day 40 | Lactobacillaceae | 0.004979835 | 1 |
| TNFa | Day 40 | Leptotrichiaceae |  | 1 |
| TNFa | Day 40 | Leuconostocaceae |  | 1 |
| TNFa | Day 40 | Micrococcaceae | -0.01721799 | 1 |
| TNFa | Day 40 | mitochondria |  | 1 |
| TNFa | Day 40 | Mogibacteriaceae |  | 1 |
| TNFa | Day 40 | Moraxellaceae |  | 1 |
| TNFa | Day 40 | Neisseriaceae | -0.074480679 | 1 |
| TNFa | Day 40 | Odoribacteraceae | 0.111907065 | 1 |
| TNFa | Day 40 | Other | 0.376756946 | 0.178142527 |
| TNFa | Day 40 | Other | 0.312973268 | 0.475047065 |
| TNFa | Day 40 | Other | 0.098946705 | 1 |
| TNFa | Day 40 | Other | 0.111907065 | 1 |
| TNFa | Day 40 | Other | 0.174077656 | 1 |
| TNFa | Day 40 | Paenibacillaceae |  | 1 |
| TNFa | Day 40 | Paraprevotellaceae |  | 1 |
| TNFa | Day 40 | Pasteurellaceae | 0.048629242 | 1 |
| TNFa | Day 40 | Peptococcaceae |  | 1 |
| TNFa | Day 40 | Peptostreptococcaceae | 0.495501358 | 0.021525152 |
| TNFa | Day 40 | Planococcaceae | -0.002482689 | 1 |
| TNFa | Day 40 | Porphyromonadaceae | -0.048881028 | 1 |
| TNFa | Day 40 | Prevotellaceae |  | 1 |
| TNFa | Day 40 | Pseudomonadaceae |  | 1 |
| TNFa | Day 40 | RFP12 |  | 1 |
| TNFa | Day 40 | Rikenellaceae |  | 1 |
| TNFa | Day 40 | Ruminococcaceae | 0.306022842 | 0.475047065 |
| TNFa | Day 40 | Spirochaetaceae |  | 1 |
| TNFa | Day 40 | Staphylococcaceae | 0.179545296 | 1 |
| TNFa | Day 40 | Streptococcaceae | 0.10525521 | 1 |
| TNFa | Day 40 | Succinivibrionaceae |  | 1 |
| TNFa | Day 40 | Tissierellaceae | -0.057435802 | 1 |
| TNFa | Day 40 | Turicibacteraceae |  | 1 |
| TNFa | Day 40 | Veillonellaceae | 0.171761887 | 1 |
| TNFa | Day 40 | Verrucomicrobiaceae | 0.111907065 | 1 |
| TNFa | Day 40 | Xanthomonadaceae |  | 1 |
| TNFa | Day 60C | Actinomycetaceae | -0.311380732 | 0.50001832 |
| TNFa | Day 60C | Aerococcaceae |  | 1 |
| TNFa | Day 60C | Aeromonadaceae |  | 1 |
| TNFa | Day 60C | Alcaligenaceae | 0.005557232 | 1 |
| TNFa | Day 60C | Bacillaceae |  | 1 |
| TNFa | Day 60C | Bacteroidaceae | 0.011196119 | 1 |
| TNFa | Day 60C | Barnesiellaceae |  | 1 |
| TNFa | Day 60C | Bifidobacteriaceae | -0.546390082 | 0.00468559 |
| TNFa | Day 60C | BS11 |  | 1 |
| TNFa | Day 60C | Burkholderiaceae |  | 1 |
| TNFa | Day 60C | Campylobacteraceae |  | 1 |
| TNFa | Day 60C | Carnobacteriaceae | 0.019326971 | 1 |
| TNFa | Day 60C | Christensenellaceae | 0.019326971 | 1 |
| TNFa | Day 60C | Clostridiaceae | 0.524796403 | 0.006622948 |
| TNFa | Day 60C | Comamonadaceae | -0.267855642 | 0.665096836 |
| TNFa | Day 60C | Coriobacteriaceae | -0.22300586 | 0.952877108 |
| TNFa | Day 60C | Corynebacteriaceae | 0.113473821 | 1 |
| TNFa | Day 60C | Dermabacteraceae |  | 1 |
| TNFa | Day 60C | Desulfovibrionaceae | -0.296110629 | 0.516587539 |
| TNFa | Day 60C | Dietziaceae |  | 1 |
| TNFa | Day 60C | Enterobacteriaceae | 0.602707572 | 0.000970546 |
| TNFa | Day 60C | Enterococcaceae | 0.078737548 | 1 |
| TNFa | Day 60C | Erysipelotrichaceae | 0.145262471 | 1 |
| TNFa | Day 60C | Eubacteriaceae | -0.057980913 | 1 |
| TNFa | Day 60C | Fibrobacteraceae |  | 1 |
| TNFa | Day 60C | Flavobacteriaceae |  | 1 |
| TNFa | Day 60C | Fusobacteriaceae | 0.264135272 | 0.665096836 |
| TNFa | Day 60C | Gemellaceae | -0.085315722 | 1 |
| TNFa | Day 60C | Helicobacteraceae |  | 1 |
| TNFa | Day 60C | Lachnospiraceae | -0.169536447 | 1 |
| TNFa | Day 60C | Lactobacillaceae | -0.109956431 | 1 |
| TNFa | Day 60C | Leptotrichiaceae |  | 1 |
| TNFa | Day 60C | Leuconostocaceae |  | 1 |
| TNFa | Day 60C | Micrococcaceae | -0.156669469 | 1 |
| TNFa | Day 60C | mitochondria |  | 1 |
| TNFa | Day 60C | Mogibacteriaceae | 0.019326971 | 1 |
| TNFa | Day 60C | Moraxellaceae |  | 1 |
| TNFa | Day 60C | Neisseriaceae | -0.22548133 | 0.952877108 |
| TNFa | Day 60C | Odoribacteraceae | 0.019326971 | 1 |
| TNFa | Day 60C | Other | 0.417079044 | 0.101140225 |
| TNFa | Day 60C | Other | -0.11104161 | 1 |
| TNFa | Day 60C | Other | -0.02359636 | 1 |
| TNFa | Day 60C | Other |  | 1 |
| TNFa | Day 60C | Other |  | 1 |
| TNFa | Day 60C | Paenibacillaceae |  | 1 |
| TNFa | Day 60C | Paraprevotellaceae |  | 1 |
| TNFa | Day 60C | Pasteurellaceae | -0.014066266 | 1 |
| TNFa | Day 60C | Peptococcaceae |  | 1 |
| TNFa | Day 60C | Peptostreptococcaceae | 0.188350103 | 1 |
| TNFa | Day 60C | Planococcaceae | 0.032480451 | 1 |
| TNFa | Day 60C | Porphyromonadaceae | -0.306526871 | 0.50001832 |
| TNFa | Day 60C | Prevotellaceae | 0.099255903 | 1 |
| TNFa | Day 60C | Pseudomonadaceae |  | 1 |
| TNFa | Day 60C | RFP12 |  | 1 |
| TNFa | Day 60C | Rikenellaceae |  | 1 |
| TNFa | Day 60C | Ruminococcaceae | -0.066950436 | 1 |
| TNFa | Day 60C | Spirochaetaceae |  | 1 |
| TNFa | Day 60C | Staphylococcaceae | 0.327865706 | 0.489128127 |
| TNFa | Day 60C | Streptococcaceae | 0.076249899 | 1 |
| TNFa | Day 60C | Succinivibrionaceae |  | 1 |
| TNFa | Day 60C | Tissierellaceae | -0.093527025 | 1 |
| TNFa | Day 60C | Turicibacteraceae |  | 1 |
| TNFa | Day 60C | Veillonellaceae | 0.184014282 | 1 |
| TNFa | Day 60C | Verrucomicrobiaceae | -0.12240415 | 1 |
| TNFa | Day 60C | Xanthomonadaceae |  | 1 |
