# Supplementary material for: Colonization by B. infantis EVC001 modulates enteric inflammation in exclusively breastfed infants
Source: Pediatr Res. 2019 Aug 23;86(6):749–57. doi: 10.1038/s41390-019-0533-2 (PMC6887859; doi:10.1038/s41390-019-0533-2)
Supplement: Supplementary file 5 — Supplementary Figure Legends [file 41390_2019_533_MOESM5_ESM.docx]

Supplemental Figure 1. **Relative abundance of specific gut taxa postnatally.** Box plots represent top 10 most abundant gut taxa for control and EVC001-fed infants at (a) Day 6, (b) Day 40, (c) Day 60. *P-*values were considered to be statistically significantly increased if *** *P < 0.05; ** P < 0.01; *** P < 0.001*; ***** P < 0.0001*** and significantly decreased if **^ *P < 0.05,* ^^ *P < 0.01;* ^^^ *P < 0.001;* ^^^^ *P < 0.0001****.*

Supplemental Figure 2. **Fecal cytokine concentrations change postnatally.** Box plots represent fecal proinflammatory cytokine concentrations [pg/mg] from the controls (n=20) and EVC001-fed infants (n=20) at Day 6 (baseline), Day 40, and Day 60 for (a) IL-2, (b) IL-5, (c) IL-6, (d) IL-8, (e) IL-10, (f) IL-22, (g) TNFα, (h) IL1β, and (i) IFNγ. Cytokine concentrations were measured in duplicate using MesoScale Discoveries U-plex. Statistical analysis was completed using Wilcoxon rank sum test. *P-*values were adjusted using the Bonferroni-Holm method and considered to be statistically significantly increased if *** *P < 0.05; ** P < 0.01; *** P < 0.001*; ***** P < 0.0001*** and significantly decreased if **^ *P < 0.05;* ^^ *P < 0.01;* ^^^ *P < 0.001;* ^^^^ *P < 0.0001****.*

Supplemental Figure 3. **Procrustes analysis of gut taxa and global cytokine profile.** Spheres represent individual samples, and they are shown to be either microbiome or global cytokine profile by being connected by a blue or red line, respectively. Connection between the spheres represent microbiomes and global cytokine profiles from the same sample and the distance between them.
